# The CovRS Environmental Sensor Directly Controls the ComRS Signaling System To Orchestrate Competence Bimodality in Salivarius Streptococci

**DOI:** 10.1128/mbio.03125-21

**Published:** 2022-01-04

**Authors:** Adrien Knoops, Florence Vande Capelle, Laetitia Fontaine, Marie Verhaegen, Johann Mignolet, Philippe Goffin, Jacques Mahillon, Andrea Sass, Tom Coenye, Laura Ledesma-García, Pascal Hols

**Affiliations:** a Louvain Institute of Biomolecular Science and Technology, Biochemistry and Genetics of Microorganisms, Université catholique de Louvain, Louvain-La-Neuve, Belgium; b Cellular and Molecular Microbiology Laboratory, Université Libre de Bruxelles, Gosselies, Belgium; c Earth and Life Institute, Laboratory of Food and Environmental Microbiology, Université catholique de Louvain, Louvain-la-Neuve, Belgium; d Laboratory of Pharmaceutical Microbiology, Ghent University, Ghent, Belgium; KUMC

**Keywords:** cell-to-cell communication, quorum sensing, bimodality, two-component system, ComRS, XIP pheromone, stochasticity

## Abstract

In bacteria, phenotypic heterogeneity in an isogenic population compensates for the lack of genetic diversity and allows concomitant multiple survival strategies when choosing only one is too risky. This powerful tactic is exploited for competence development in streptococci where only a subset of the community triggers the pheromone signaling system ComR-ComS, resulting in a bimodal activation. However, the regulatory cascade and the underlying mechanisms of this puzzling behavior remained partially understood. Here, we show that CovRS, a well-described virulence regulatory system in pathogenic streptococci, directly controls the ComRS system to generate bimodality in the gut commensal Streptococcus salivarius and the closely related species Streptococcus thermophilus. Using single-cell analysis of fluorescent reporter strains together with regulatory mutants, we revealed that the intracellular concentration of ComR determines the proportion of competent cells in the population. We also showed that this bimodal activation requires a functional positive-feedback loop acting on ComS production, as well as its exportation and reinternalization via dedicated permeases. As the intracellular ComR concentration is critical in this process, we hypothesized that an environmental sensor could control its abundance. We systematically inactivated all two-component systems and identified CovRS as a direct repression system of *comR* expression. Notably, we showed that the system transduces its negative regulation through CovR binding to multiple sites in the *comR* promoter region. Since CovRS integrates environmental stimuli, we suggest that it is the missing piece of the puzzle that connects environmental conditions to (bimodal) competence activation in salivarius streptococci.

## INTRODUCTION

The asexual mode of proliferation in bacteria is one of the most successful strategies in living organisms but comes with the cost of low genetic diversity (1). To overcome this drawback, bacteria have evolved complex horizontal gene transfer mechanisms such as conjugation and natural transformation. Those processes are extremely efficient to generate genomic diversity and allow integration of DNA derived not only from closely related species but also from distant clades (1, 2). Recently, accumulation of genomic sequencing data has underlined the importance of those mechanisms for bacterial adaptation and evolution (3).

In bacteria, natural DNA transformation is triggered upon activation of a master transcriptional regulator and subsequent entry into a transient physiological state called competence (3). Mechanisms of competence activation and regulation can drastically differ, even between closely related bacterial species. However, the cost of this strategy in terms of energy and metabolism shifting is significant (4, 5), and as a consequence, activating this process can be fateful. To share responsibility at the population scale, initiating the system only in a subset of the community (bet-hedging) is a useful strategy, and this is presumably why bacteria have evolved complex regulatory pathways to control competence development. Optimally benefiting from biological noise, these regulatory systems can stochastically activate the switch from the noncompetent to competent state (6). Stochastic cellular decision-making processes promoting heterogeneous competence activation have been thoroughly studied in Bacillus subtilis (7, 8). It has been determined in this species that the main factor for competence development relies on the master regulator ComK (9). By experimentally manipulating the level of noise, it has been shown that a twofold decrease of noise in *comK* expression led to a 10-fold reduction of the probability for switching to the competence state (7). A critical aspect is the temporal modulation of the basal level of ComK that transiently pushes individual cells to reversibly switch from the noncompetent to the competent state. Indeed, bimodal cell-to-cell distribution of molecules like ComK is often referred as a bistable system. However, competence development should be considered an excitable process rather than a classical bistable system as it stochastically switches on in a subpopulation for a short period of time (8).

**FIG 1:**
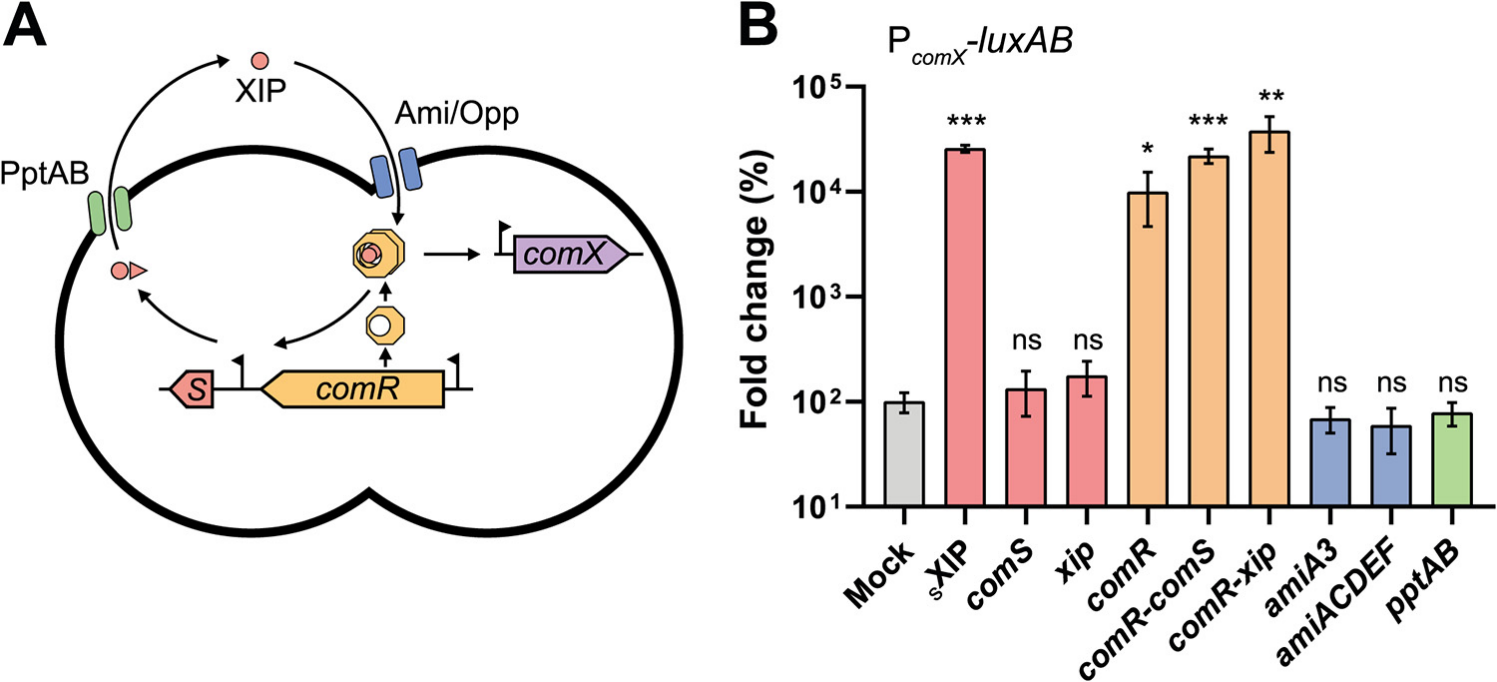
Analysis of ComRS bottleneck players. (A) Scheme of the ComRS system showing all components that were overexpressed. (B) Fold change of maximum specific luciferase activity (relative light units [RLU]/OD_600_) for the overexpression of the different ComRS components compared to mock strain (P*_comX_-luxAB* reporter strain). For induction, 1% xylose and 1 mM IPTG were added for the xylose-inducible promoter (*comS*, *xip*, *comR*, *comR-comS*, *comR-xip*, *amiACDEF*) and the lactose-inducible promoter (*pptAB*), respectively. For synthetic XIP induction (sXIP), a 500 nM concentration of the peptide was added. Values are mean values (*n *= 3) ± standard deviations (error bars). One-way ANOVA with Dunett’s test was performed for each overexpressed component in comparison to mock strain (ns, not significant; *, *P* < 0.05; **, *P* < 0.01; ***, *P* < 0.001).

In streptococci, two main cell-to-cell communication systems for the control of competence have been described, ComCDE for the mitis and anginosus groups and ComRS for all other groups. While both systems show common features such as a transcriptional positive feedback on pheromone production, heterogeneous activation of competence has been mostly investigated for ComRS-containing species (10–15). The latter system involves two early competence (*com*) genes responsible for the induction of the master regulator ComX (alternative σ^X^ factor) and subsequent activation of late *com* genes required for the transformasome assembly (Fig. 1A) (16–19). More precisely, ComX induction is triggered by the small peptide XIP (*comX-*inducing peptide) acting as a signaling pheromone. This oligopeptide is the mature form of an intracellular precursor (ComS), which can be exported by the PptAB transporter and processed by the Eep peptidase as shown in Streptococcus thermophilus (17, 20) (Fig. 1A). Subsequently, XIP is reimported through the Ami/Opp transporter and binds to the transcriptional sensor ComR. This binding triggers ComR dimerization, resulting in the formation of the ComR·XIP complex that amplifies the early competence stage by enhancing *comS* transcription through a positive-feedback loop (19, 21). Above a specific XIP concentration, ComR·XIP also induces ComX accumulation; ComX subsequently binds to the RNA polymerase and triggers the transcription of late *com* genes (Fig. 1A). In Streptococcus salivarius, the ComRS system additionally couples the activation of competence to predatory mechanisms (22), presumably to ensure the release of free DNA from bacteriocin-sensitive preys.

Understanding phenotypic heterogeneity and answering the question of how the ComRS system is able to generate a bimodal activation of competence in streptococci have remained challenging. This phenomenon has been mostly studied in Streptococcus mutans, where competence is regulated by both ComRS and the transcriptional regulation system BlpRH, ambiguously named ComCDE (10, 11). In this species, bimodality arises only via the activation of BlpRH through its cognate peptide MIP (mutacin-inducing peptide) but requires the ComRS system, suggesting a ComRS-triggering role for BlpRH (11, 12, 14).

In the present study, we investigated the origin of the ComRS-related bimodality in competence activation. We used S. salivarius, a model that does not contain a second competence-associated pheromone system such as the ComCDE/BlpRH found in S. mutans (22). We showed that the intracellular concentration of the sensor protein ComR is the key factor that controls the bimodal activation of competence in *S. salivarius*. Searching for a two-component system (TCS) controlling *comR* expression, we unexpectedly revealed that CovRS, a well-described virulence-controlling system in streptococci, governs the amount of ComR and thus competence bimodality in salivarius streptococci.

## RESULTS

### Competence activation is ComR sensitive.

Recently, we showed that the human gut-isolated *S. salivarius* HSISS4 strain was able to activate natural transformation through the ComRS system upon addition of the synthetic mature form of ComS (sXIP) (22). To unveil activation bottlenecks in the ComRS pathway, we overexpressed all known components of the system (Fig. 1A). For this purpose, we first transformed the *comX* promoter to luciferase reporter genes (P*_comX_*-*luxAB*) into an ectopic locus in the HSISS4 strain. We then used this competence reporter strain to construct transcriptional fusions of the different known components of the ComRS system under the control of inducible or strong constitutive promoters (Fig. 1B). In line with previous data (22, 23), we observed a high activation of the signaling pathway upon ComR increase (P*_xyl2_-comR*, ∼100-fold increase). In addition, we constructed *comR* overexpression systems in tandem with *comS* (full-length pheromone, P*_xyl2_-comR-comS*) or *xip* (an artificial gene encoding a 12-amino-acid [aa] intracellular form of ComS, P*_xyl2_-comR-xip*). Those strains showed high reactivity upon transcriptional induction (220- and 380-fold increase, respectively), presumably through bypassing *comS* transcriptional activation by ComRS and pheromone export-import, respectively (Fig. 1B). Although their functionality was validated (see Fig. S1A and B in the supplemental material), upregulation of the PptAB pheromone exporter (20) (P*_lac_-pptAB*) or importer (16) (AmiA3, P_32_*-amiA3* or AmiACDEF, P*_xyl2_-amiACDEF*) showed no increase in competence activation. Unexpectedly, *comS* (P*_xyl2_-comS*) or *xip* (P*_xyl2_-xip*) overexpression had no effect on P*_comX_*, while exogenous sXIP addition elicited a marked response (260-fold increase) (Fig. 1B). Because functionality of the two constructs was confirmed (Fig. S1C), we concluded that the system was not sensitive to endogenous pheromone increase at native ComR concentration. Together, these results demonstrate that competence acquisition in *S. salivarius* depends mainly on the amount of ComR.

10.1128/mBio.03125-21.1 FIG S1 Validation of overexpression fusions. Luciferase and GFP microscope assays were performed in order to show the functionality of different overexpression constructs unable to activate competence (Fig. 1). (A) Functionality of a P*_lac_-pptAB* (P*_comX_-gfp^+^* as proxy) conditional mutant (native locus) through its rescue by IPTG induction of P*_lac_*. Competence was activated by xylose addition (0.5%) thanks to the P*_xyl2_-comR* overexpression construct. (B) Functionality of P*_xyl2_-amiACDEF* through xylose induction (1%) in a WT P*_comX_-luxAB* reporter strain. Competence was activated by _S_XIP addition (500 nM). Results are displayed as maximum specific luminescence (RLU/OD_600_). (C) Functionality of P*_xyl2_-comS* and P*_xyl2_-xip* through complementation of a *ΔcomS* (P*_comX_-gfp^+^* as proxy) strain. Competence was activated through the P*_xyl2_-comR* overexpression construct. Overexpression fusions were induced by xylose addition (0.5%). For GFP assays (panels A and C), three populations from biological replicates were analyzed and their median fluorescence (arbitrary unit [AU]) was calculated. The mean median values of the three populations together with standard deviations are displayed. For the luciferase assay (panel B), biological triplicates were used. One-way ANOVA with Dunnett’s test was performed for multiple comparison to the mock strain (panel A) and *t* test was performed for simple comparison to related mock strain (panels B and C) (ns, not significant; **, *P* < 0.01; ***, *P* < 0.001; **** *P* < 0.0001). Download FIG S1, PDF file, 0.5 MB. Copyright © 2022 Knoops et al. 2022 Knoops et al. https://creativecommons.org/licenses/by/4.0/ This content is distributed under the terms of the Creative Commons Attribution 4.0 International license.

### ComR levels dictate bimodality.

**FIG 2:**
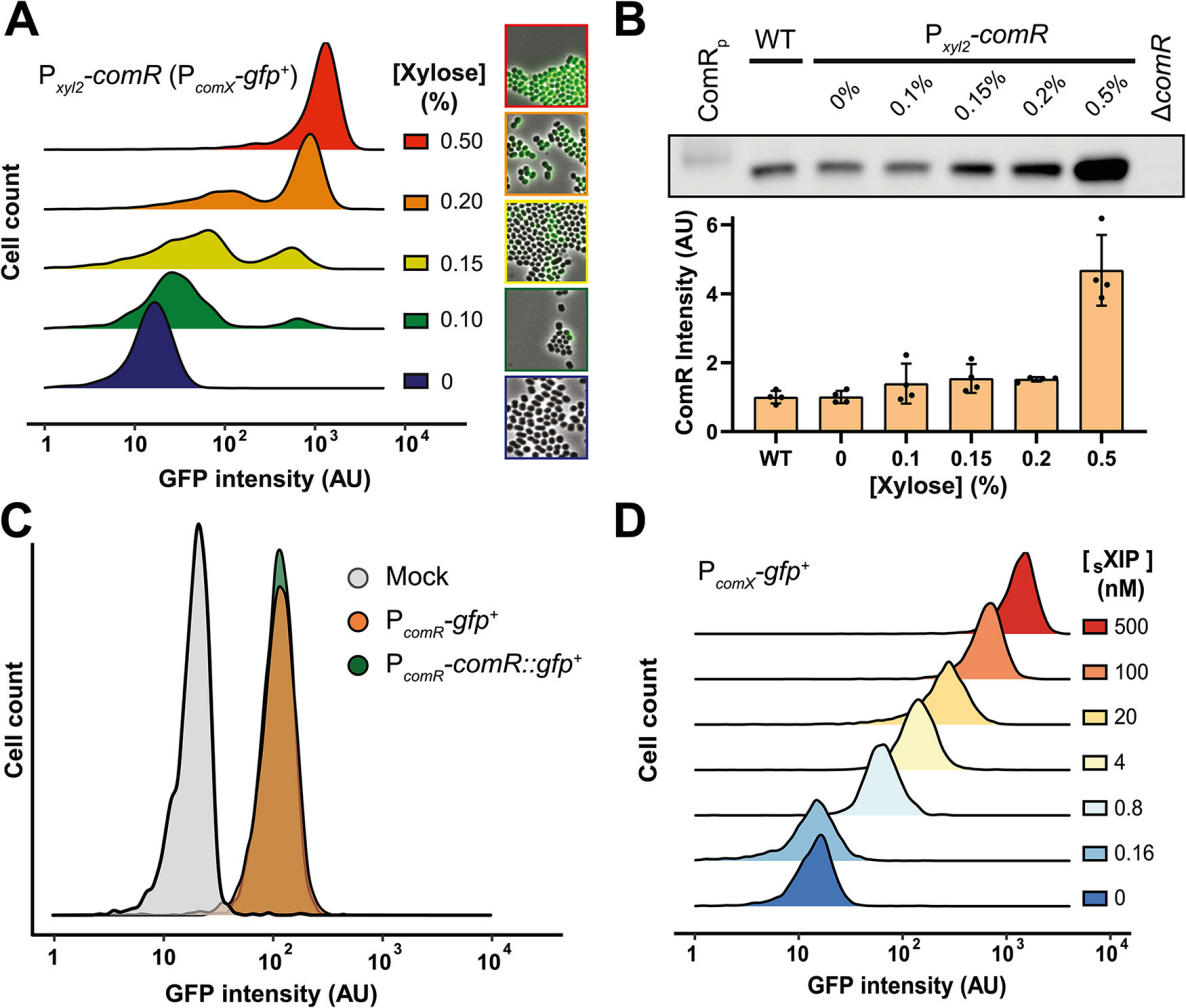
Control of competence bimodality by ComR. (A) Density plot of single-cell green fluorescent protein (GFP) intensity (arbitrary unit [AU], log scale) from a gradient of ComR levels. The overexpression of *comR* (P*_xyl2_-comR*) was monitored with a P*_comX_*-*gfp^+^* reporter strain in the presence of increasing xylose concentrations (shown as a percentage). Pictures on the right are representative cell samples of each xylose concentration tested. (B) Semiquantification of ComR by immunoblotting. ComR Western blot with crude extracts (technical replicates [*n *= 4]) from WT (strain HSISS4), Δ*comR* mutant (negative control), and *comR*-overexpressing strain (P*_xyl2_-comR* P*_comX_-gfp^+^*) incubated 2 h with increasing xylose concentrations (shown as a percentage). ComR_p_ denotes recombinant ComR-StrepTag used as positive control. Gel band intensities were quantified and normalized using total protein quantification from Coomassie blue staining. (C) Density plot of mock strain (gray), P*_comR_* activation (orange), and ComR levels (green) from microscopic analysis of HSISS4 WT, P*_comR_-gfp^+^*, and P*_comR_-comR::gfp^+^* fluorescence intensities, respectively. (D) Density plot of an sXIP dose-response (nM) from microscopic analysis of P*_comX_-gfp*^+^ fluorescence intensities. For panels A, C, and D, the fluorescence of individual cells was examined from biological triplicates (*n *> 500 cells in each experiment).

Since ComR is the most sensitive parameter for competence activation, we sought to determine its importance for ComRS-mediated competence bimodality. We therefore designed a transcriptional fusion between P*_comX_* and a 5′ untranslated region (5′-UTR)- and codon-optimized *gfp^+^* gene (P*_comX_-gfp^+^*). As *S. salivarius* is not spontaneously competent under laboratory conditions, we transferred the xylose-inducible *comR* cassette (P*_xyl2_-comR*) into this background and performed microscopic analysis with populations incubated with various xylose concentrations (Fig. 2A). Results showed the emergence of a high *gfp^+^*-expressing subpopulation proportional to xylose increase. We observed a bimodal activation of P*_comX_* from 0.1 to 0.2% xylose concentration, with 0.5% displaying unimodal P*_comX_* activation across the population (Fig. 2A). To validate that bimodality was not an artifact due to the xylose-inducible promoter itself, we constructed a P*_xyl2_-gfp^+^* strain. Cell populations of this control strain incubated with the same range of xylose concentrations exhibited no bimodal activation (Fig. S2), supporting the idea that bimodality arises from the ComRS system itself. To measure the amount of ComR protein added to the native system through the P*_xyl2_-comR* construct, we performed Western blot analyses on crude cell extracts from xylose-induced samples (Fig. 2B). Comparison with a noninduced condition showed that a small increase in the amount of ComR (<1.5-fold at the population level) was sufficient to activate P*_comX_* in a subpopulation. Finally, to assess that bimodality was arising from the topology of the ComRS regulatory circuitry and not from an upstream bimodal transcription of *comR*, we analyzed its expression at the single-cell level using a P*_comR_-gfp^+^* reporter strain. Microscopic analysis showed unimodal *gfp^+^* expression for the whole population, suggesting a low-noise *comR* transcription (Fig. 2C). To verify ComR unimodality at the translational level, we performed a population-wide microscopic analysis of a ComR-GFP^+^ fusion (P*_comR_*-*comR::gfp*^+^) (Fig. 2C) and a sXIP dose-response on P*_comX_-gfp^+^* expression (Fig. 2D). A unimodal response was observed under all tested conditions, revealing that the distribution of ComR among the cells in the population does not phenocopy the bimodal distribution of *comX* expression.

10.1128/mBio.03125-21.2 FIG S2 Absence of P*_xyl2_* intrinsic bimodality. (A) Density plot of single-cell GFP intensity (arbitrary unit [AU], log scale) from microscopic analysis of the P*_xyl2_-gfp^+^* strain incubated with 0, 0.1, 0.15, 0.2, and 0.5% of xylose. The fluorescence of individual cells was examined from biological triplicates (*n *> 500 cells in each experiment). (B) Linear regression performed on median GFP intensities of cell populations (black dots denote median values of distribution of biological replicates). Dashed lines denote confidence intervals (0.05) of the regression. Download FIG S2, PDF file, 0.6 MB. Copyright © 2022 Knoops et al. 2022 Knoops et al. https://creativecommons.org/licenses/by/4.0/ This content is distributed under the terms of the Creative Commons Attribution 4.0 International license.

Altogether, those results show that the level of ComR is important for the activation of ComRS into a hypersensitive mode and is the key factor for competence bimodal activation; the latter feature does not arise from heterogenous ComR distribution in the population.

### A positive-feedback loop is required for bimodal activation.

**FIG 3:**
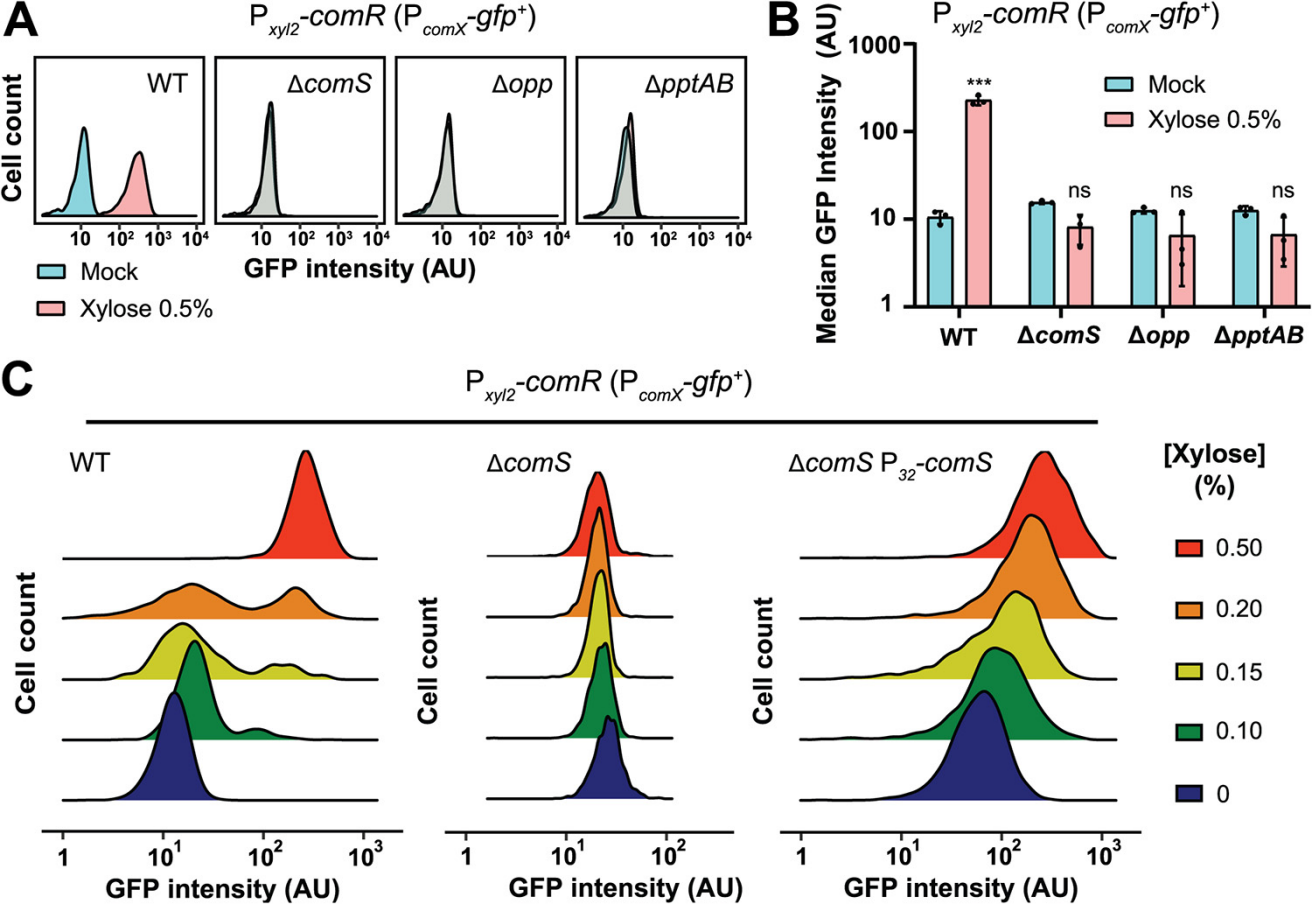
Implication of the positive loop in competence bimodality. (A) Representative density plots of loop inactivation from microscopic analysis of a P*_comX_-gfp^+^* P*_xyl_*_2_-*comR* strain harboring either a native loop (WT) or an inactivated loop through deletion of *comS* (pheromone precursor; *ΔcomS*), *amiACDEF* (XIP importer; *Δopp*), or *pptAB* (XIP exporter; *ΔpptAB*). Each subpanel depicts a population fluorescence analysis for each strain either without (mock) or with strong xylose induction (0.5%). (B) Bar chart depicting biological triplicates of the representative loop inactivation experiments shown in panel A. Values are means of median values (*n *= 3) ± standard deviations (error bars). Statistical *t* tests were performed for each inactivated component in comparison to its related mock strain (ns, not significant; ***, *P* < 0.001). (C) Representative density plot of single-cell GFP intensity (arbitrary unit [AU], log scale) from a gradient of ComR levels. The overexpression of *comR* (P*_xyl2_-comR*) was monitored with a P*_comX_*-*gfp^+^* reporter strain in the presence of increasing xylose concentrations (shown as a percentage). The same conditions were applied to three different genetic backgrounds: a mock strain (P*_comX_-gfp^+^* P*_xyl_*_2_-*comR*) and the same strain deficient for the pheromone (Δ*comS*) and with the pheromone under the control of a strong constitutive promoter, unplugged from the positive-feedback loop (Δ*comS* P*_32_-comS*). The fluorescence of individual cells was examined from biological triplicates (*n *> 500 cells in each experiment).

While we showed that ComR levels played a central role in the bimodal activation of competence, we had no information on mechanisms involved in this phenomenon. Since positive-feedback-loop-based networks have been shown to generate heterogeneity in other bacterial systems (24, 25), we investigated the role of the ComRS feedback loop for the bimodal activation of the system (Fig. 1A). For this purpose, we inactivated the loop by constructing mutants of its key players (i.e., Δ*comS*, Δ*amiACDEF*, and Δ*pptAB*) into a P*_comX_-gfp^+^* background harboring the P*_xyl2_-comR* overexpression cassette. ComR overproduction through xylose induction in those strains showed no P*_comX_* activation, underlining the necessity of the pheromone, and its export and import for ComR-mediated activation (Fig. 3A and B).

We then focused on the origin of bimodality and its link to the positive-feedback loop. For this purpose, we compared a wild-type (WT) strain to Δ*comS* and Δ*comS* P_32_-*comS* mutants (Fig. 3C). The first mutant is deficient in pheromone production, while the second expresses the pheromone under the control of a strong constitutive promoter unplugged from the feedback loop. Of note, those three strains possess the P*_xyl2_*-*comR* overexpression cassette in a P*_comX_-gfp^+^* background. We applied various xylose concentrations and observed no competence activation for the pheromone-deficient strain (Δ*comS*), while the reference strain showed bimodal activation for mild xylose induction (Fig. 3C). In contrast, the mutant producing the pheromone unwired from the loop showed a unimodal activation, gradually increasing in line with *comR* overexpression (Fig. 3C). Together, these results show that the ComRS positive-feedback loop is necessary for ComR-mediated competence activation and its bimodal behavior.

### The two-component system CovRS controls *comR* expression.

**FIG 4:**
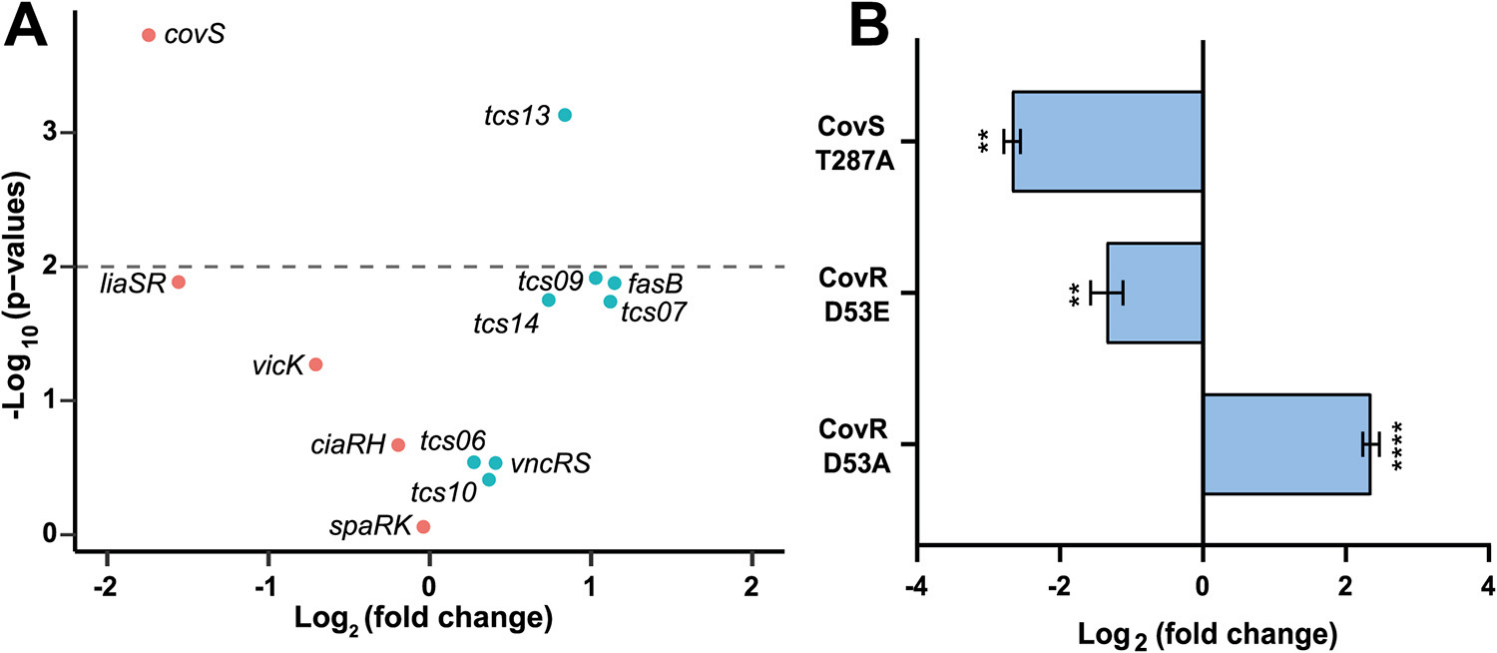
The two-component system CovRS controls *comR* expression. (A) Volcano plot showing the inactivation effect of complete TCS or their histidine kinase (*covS*, *vicK*, and *fasB*) on P*_comR_* activation. The dashed line denotes threshold corresponding to *P* = 0.01, and green and red dots show *comR* upregulation and downregulation, respectively. (B) Analysis of key residues of CovRS activity on *comR* expression. The phosphorylated residue of CovR was mutated either in alanine (D53A) or glutamate (D53E) to mimic the unphosphorylated or phosphorylated state, respectively. The CovS residue for phosphatase activity was mutated in alanine (T287A). For panels A and B, P*_comR_* activation was monitored with the P*_comR_*-*luxAB* reporter strain. Maximum specific luciferase activity (RLU/OD_600_, log_2_) were computed from biological triplicates, and mean values were used to calculate fold changes (± standard deviation in panel B) in comparison to mock strain. One-way ANOVA with Dunnett’s test was performed on log_2_ fold change data to generate *P* value*s* (**, *P* < 0.01; ****, *P* < 0.0001 in panel B).

As we identified that the amount of ComR is central in the bimodal activation of competence, we investigated how ComR production and subsequent bimodality were controlled. Various two-component systems (TCSs) that sense environmental signals were previously shown to modulate competence in streptococci (26–28), and we decided to investigate their role in *comR* regulation. To this end, we conducted an *in silico* search in the HSISS4 genome and identified 13 putative TCSs harboring a response regulator in tandem with a histidine kinase (see Table S1 in the supplemental material). To investigate their role in *comR* regulation, we successfully inactivated 10 complete TCSs in the P*_comR_-luxAB* reporter strain (Fig. 4A). Because we were not able to generate three mutants (Δ*covRS*, Δ*fasAB*, and Δ*vicRK*), presumably due to their essential functions, we generated viable single gene deletions of their histidine kinase (Δ*covS*, Δ*fasB*, and Δ*vicK*). Since the Δ*covS* mutant showed the strongest and most significant effect on *comR* expression (Fig. 4A), we decided to investigate the importance of the CovRS system in more depth.

10.1128/mBio.03125-21.7 TABLE S1 Annotation of TCSs from *S. salivarius* HSISS4. Download Table S1, PDF file, 0.1 MB. Copyright © 2022 Knoops et al. 2022 Knoops et al. https://creativecommons.org/licenses/by/4.0/ This content is distributed under the terms of the Creative Commons Attribution 4.0 International license.

The CovRS system (control of virulence) has been well characterized in Streptococcus pyogenes and Streptococcus agalactiae where it regulates the expression of a high proportion of the genome (∼10%), including several virulence genes (29, 30). Mechanistic studies have shown that upon activation through a stimulus (e.g., human defensin, divalent cations, or osmotic shock), CovS is able to trigger either phosphorylation (via a glutamate residue) or dephosphorylation (via a threonine residue) of an aspartate on its cognate response regulator CovR (31–34). As a result of phosphorylation, CovR forms dimers and binds DNA, mainly as a repressor (35). Because we identified a conserved motif in CovS at the phosphatase site, we decided to perform a point mutation and substitute the key T287 residue for alanine. In parallel, we produced two other point mutations of the phosphorylation site of CovR, D53A and D53E, mimicking the unphosphorylated and constitutive phosphorylated state, respectively. We transferred those mutations into a P*_comR_-luxAB* background and monitored the fold change in expression (Fig. 4B). Because CovR_D53E_ and CovS_T287A_ overrepressed *comR* expression while CovR_D53A_ relaxed repression, we concluded that the phosphorylated form of CovR (CovR∼P) is responsible for *comR* repression. In addition, transcriptome sequencing (RNA-seq) experiments (Table S2) showed that CovS_T287A_ and CovR_D53E_ mutants overrepressed most of the genes of the ComR regulon that was previously established, as well as a large set of genes directly controlled by ComX which are essential for natural transformation (22).

10.1128/mBio.03125-21.8 TABLE S2 Down-regulated genes in CovS_T287A_ and CovR_D53E_ mutants belonging to the ComR regulon. Download Table S2, PDF file, 0.2 MB. Copyright © 2022 Knoops et al. 2022 Knoops et al. https://creativecommons.org/licenses/by/4.0/ This content is distributed under the terms of the Creative Commons Attribution 4.0 International license.

Altogether, these data demonstrate that the CovRS system is pivotal in the control of competence development in *S. salivarius* through modulation of *comR* expression.

### CovR inhibits *comR* expression through direct transcriptional control.

**FIG 5:**
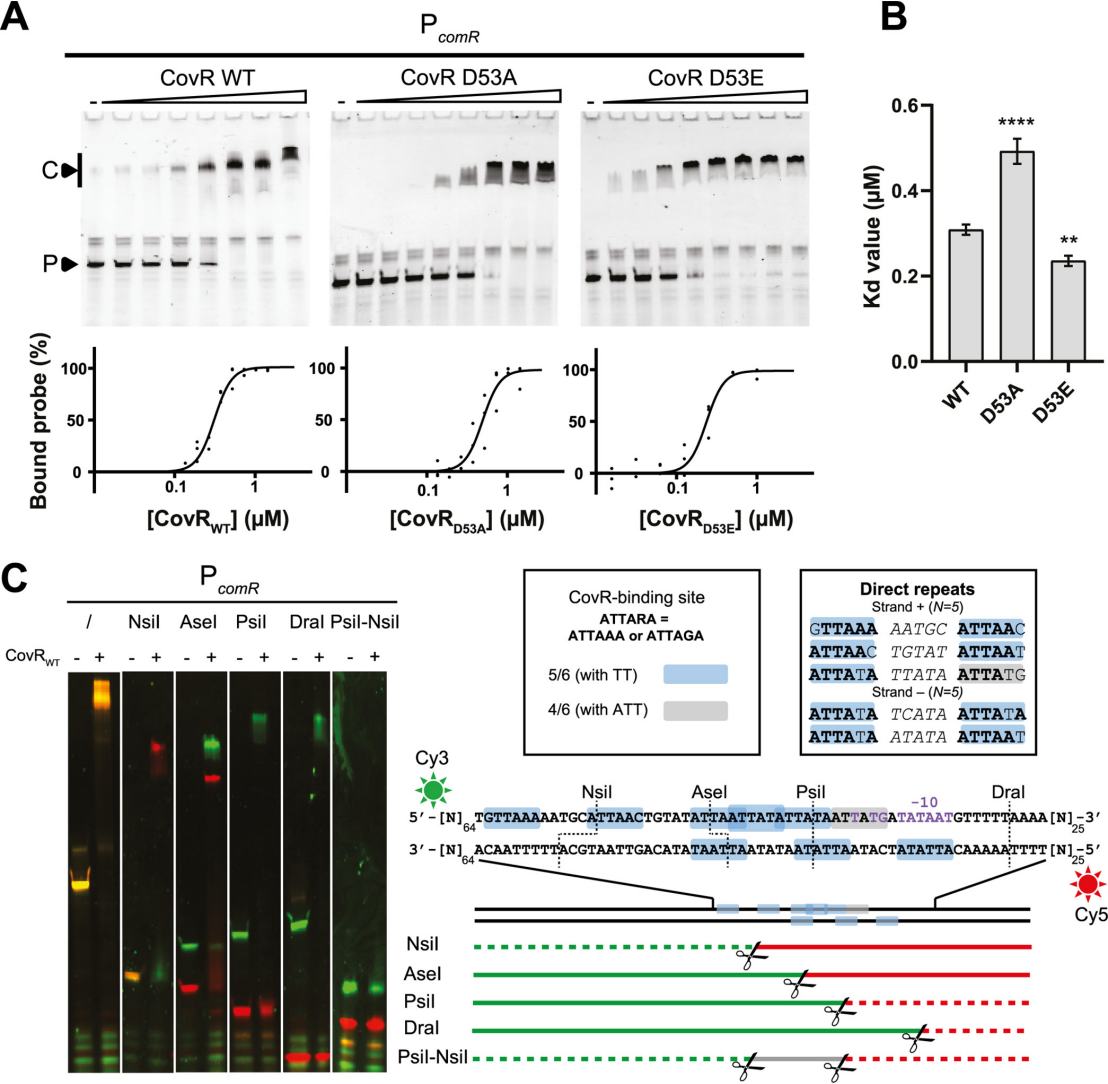
Characterization of CovR binding to P*_comR_*. (A) Representative EMSAs (top panels) performed with the P*_comR_* probe (150 bp, 0.05 μM) and a gradient of purified CovR_WT_, CovR_D53A_, or CovR_D53E_ (triangles, maximum of 1.4 μM with serial 1.4:1.4 dilutions). Lanes with no CovR are indicated by a minus sign. P and C denote the unbound probe and the CovR-DNA complex, respectively. Gels displayed are representative of technical triplicates. Nonlinear regressions of percentage of bound probes (black dots show triplicates values) were implemented using a Hill equation (*n *= 4) (lower panels). (B) Comparison of estimated *K_d_* values (± standard deviation) between CovR variants. These values were extrapolated from nonlinear regressions shown in panel A. One-way ANOVA with Dunnett’s test was performed for the *K_d_* values of CovR variants in comparison to WT (*, *P* < 0.05; **, *P* < 0.01, *n* = 3). (C) Mapping of CovR binding motifs by restriction of the P*_comR_* probe. EMSA experimental data and schematic view of P*_comR_* restriction pattern are displayed on left and right, respectively. The P*_comR_* probe (150 bp) was double labeled with Cy3 and Cy5 fluorophores and restricted by NsiI, AseI, PsiI, DraI, and PsiI-NsiI for the fine mapping of CovR binding motifs. The resulting products (0.05 μM or 0.1 μM for unrestricted and restricted DNA, respectively) were incubated with (+) or without (−) CovR_WT_ (0.43 μM). The depicted double-stranded DNA sequence shows positions 65 to 125 of the P*_comR_* probe. Putative CovR-binding sites (ATTARA) upstream of the predicted −10 box (purple) of P*_comR_* are highlighted by colored boxes (light blue, one mismatch with TT conservation; gray, two mismatches with ATT conservation). Direct repeats separated by 5 nucleotides are highlighted in a separate box. Green and red sun icons represent 5′-end Cy3 and 3′-end Cy5 labeling, respectively. The positions of scissors denote cleavage sites of restriction enzymes, and green/red horizontal lines display restriction products with color code corresponding to their labeled fluorophore. Continuous and dashed lines denote presence and absence of a shift on the gel, respectively.

As the CovRS system had shown strong control over the *comR* promoter *in vivo*, we characterized *in vitro* interactions between CovR and P*_comR_*. To this end, we purified three variants of CovR (CovR_WT_, CovR_D53A_, and CovR_D53E_) and performed electrophoretic mobility shift assays (EMSAs) on Cy3-labeled DNA corresponding to P*_comR_* (150 bp upstream of the start codon) (Fig. 5A and B). The results showed binding of purified CovR_WT_ to P*_comR_*, which is lower and higher for the CovR_D53A_ variant and the phosphomimetic CovR_D53E_, respectively. The approximately twofold difference in binding efficiency between the two CovR mutants is similar to previously reported data between unphosphorylated CovR and CovR∼P from other streptococci (36). Although this difference seems low, it has been demonstrated that the presence of the RNA polymerase enhances the CovR∼P repression, explaining higher *in vivo* repression (35). To demonstrate the specificity of this binding, we performed EMSA with the promoter and an internal coding sequence (CDS) fragment of the physiologically unrelated gene *dnaE* (alpha subunit of polymerase III [PolIII], P*_dnaE_* and CDS*_dnaE_*, respectively), as well as the promoter of CovR (P*_covR_*) that has previously been reported to be bound by CovR in other streptococci (37, 38). While CovR showed binding to P*_covR_*, weak unspecific binding at very high CovR concentrations and no binding were observed for P*_dnaE_* and CDS*_dnaE_*, respectively (Fig. S3A). Furthermore, competition assays with unlabeled probes (P*_comR_*, P*_dnaE_*, and CDS*_dnaE_*) showed a decrease in CovR-P*_comR_* interaction for the P*_comR_* competing probe but no effect on P*_dnaE_* and CDS*_dnaE_*, underlining the specificity of these interactions (Fig. S3B).

10.1128/mBio.03125-21.3 FIG S3 Specificity controls for P*_comR_-*CovR interaction. (A) EMSA with nonspecific (P*_dnaE_* and CDS*_dnaE_*) and specific (P*_covR_*) probes (150 bp, 0.05 μM) and a gradient of purified CovR_D53A_ or CovR_D53E_ (triangles, maximum of 1.4 μM with 1.4×  serial dilutions). (B) EMSA competition assays performed between the P*_comR_-*Cy3 (150 bp, 0.05 μM)-CovR_D53E_ (0.7 μM) complex and a gradient of unlabeled nonspecific probes (P*_dnaE_* and CDS*_dnaE_*) or specific probe (P*_comR_*) (triangles, maximum of 2 μM with 1:2 serial dilutions). In panels A and B, minus and plus signs depict the absence and presence of unlabeled probe or protein, respectively. P and C denote the unbound probe and the CovR-DNA complex, respectively. Download FIG S3, PDF file, 2.6 MB. Copyright © 2022 Knoops et al. 2022 Knoops et al. https://creativecommons.org/licenses/by/4.0/ This content is distributed under the terms of the Creative Commons Attribution 4.0 International license.

Previous work with S. pyogenes had identified the DNA motif responsible for CovR binding (ATTARA motif) (36, 37), and we screened the *comR* promoter for this motif (Fig. 5C). We found many motifs with one mismatch but no consensus sequences. Particularly, the region just upstream of the predicted −10 box showed a high density of directly repeated motifs on both DNA strands, which in most cases are separated by five nucleotides (Fig. 5C). To determine whether that region could bind CovR, we performed EMSA with a rational restriction of the P*_comR_* probe labeled with Cy3 and Cy5 at the 5’and 3′ end, respectively (Fig. 5C). While unrestricted P*_comR_* showed an orange band shift upon CovR binding due to red and green fluorophore colabeling, the restriction mapping based on the mobility shift of 5′-end (green) and 3′-end (red) labeled fragments showed that only the region upstream of the −10 box with the mapped motifs is able to bind CovR (Fig. 5C). Particularly, we observed a shift for both 5′ and 3′ fragments when restriction was performed in the middle of the binding region (AseI), suggesting interaction of CovR with multiple sites. In addition, a double restriction surrounding this region (PsiI-NsiI) showed no band shift, which excludes the presence of other binding sites in the 150-bp fragment upstream of the start codon of *comR*.

Altogether, these results suggest that the CovRS system controls competence activation in *S. salivarius* by directly repressing *comR* transcription through binding of CovR to multiple sites in its promoter region.

### CovR represses competence in the salivarius group.

**FIG 6:**
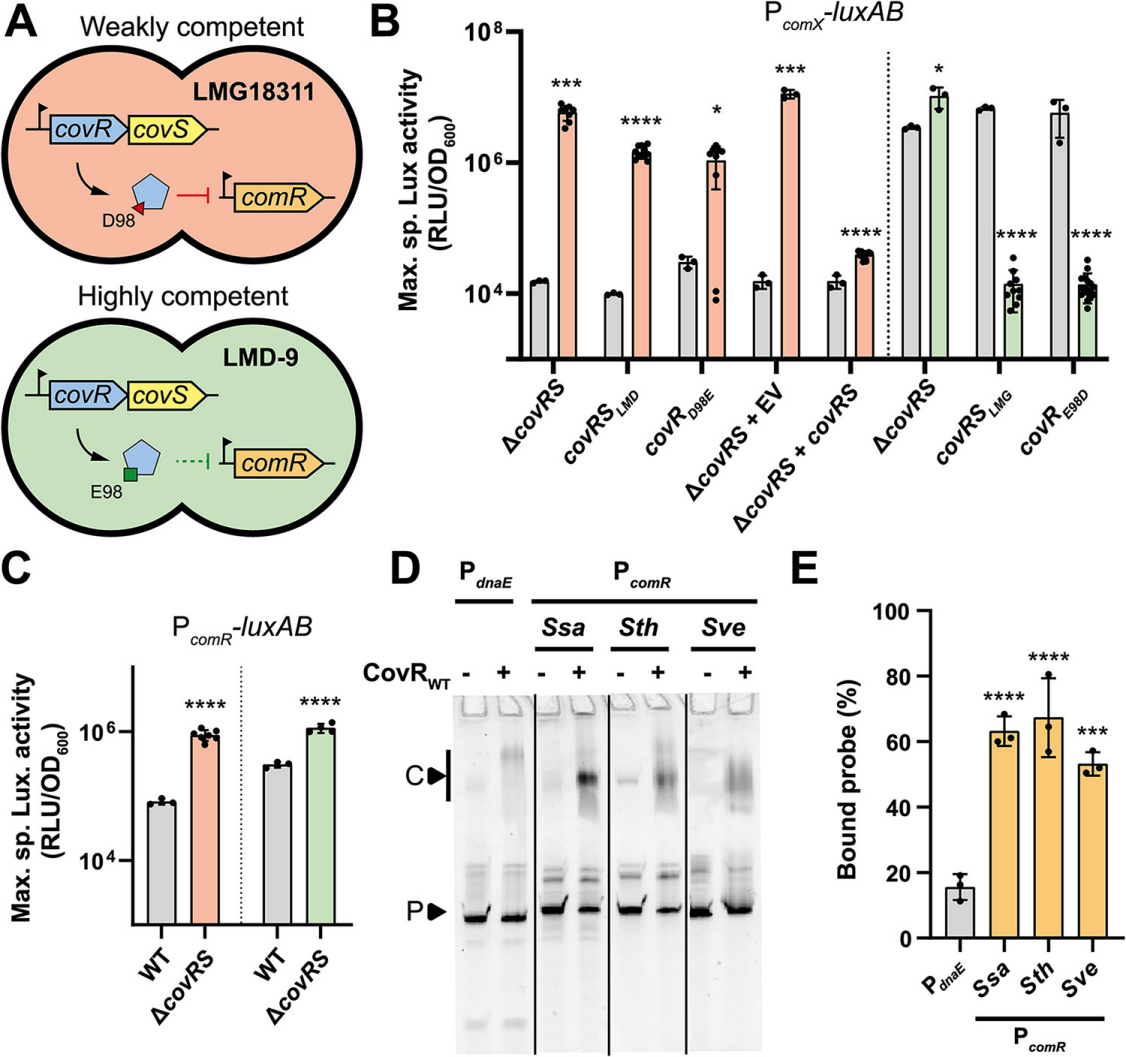
Distal regulation of ComR by CovRS in salivarius streptococci. (A) Schematic view of the two CovRS modules present in strains LMG18311 (orange) and LMD-9 (green) of S. thermophilus. Red and green lines illustrate strong and weak repression effect of CovRS on P*_comR_*, respectively. The weak repression effect in LMD-9 is due to CovR_D98E_ mutation, which correlates with its high spontaneous transformability. (B and C) Effect of CovRS modulation on P*_comX_*/P*_comR_* activity (B/C) in strains LMG18311 (orange) and LMD-9 (green). Data show maximum specific luciferase activity (RLU/OD_600_) for Δ*covRS* mutants, *covRS* reciprocal swap (LMG/LMD), *covR* point mutation exchange (D98E/E98D), plasmid complementation of LMG18311 Δ*covRS* (Δ*covRS* plus *covRS*) and its control (Δ*covRS* plus empty vector [EV]) in comparison to their related control strain (gray). The control strains are LMG18311 WT or LMD-9 WT, except for LMG18311 complementation experiments where the WT strain contains the empty vector. (D) Representative EMSA performed with the negative-control P*_dnaE_* or P*_comR_* probes of *S. salivarius* (*Ssa*) HSISS4, S. thermophilus (*Sth*) LMD-9, and *S. vestibularis* (*Sve*) NCTC12167 (150 bp, 0.05 μM) in the presence (+) or absence (−) of CovR_WT_ from *Ssa* HSISS4 (0.4 μM). (E) Bar chart showing the percentage of bound probe for each promoter shown in panel C. Dots show the values for biological replicates (panels B and C, *n *≥ 3) or technical triplicates (panel E) ± standard deviations. Statistical *t* test was performed for each condition in comparison to related mock strain (WT: mock, panels B and C) and one-way ANOVA with Dunnett’s test was performed for multiple comparison (P*_dnaE_*: mock, panel E) (*, *P* < 0.05; ***, *P* < 0.001; ****, *P* < 0.0001).

We next investigated whether the CovRS-mediated competence repression could be broadened to other streptococci of the salivarius group. Since inactivation of the complete CovRS system in the domesticated species S. thermophilus was previously reported as nonlethal (39), we deleted *covRS* in luminescence reporter strains of P*_comX_*/P*_comR_* activity. S. thermophilus strains LMG18311 and LMD-9 were compared based on their reported transformability in the absence of sXIP addition, which is very low and high, respectively (40). CovRS inactivation showed that the CovR-mediated repression of competence was also operational on the control of P*_comR_* and P*_comX_* in S. thermophilus (Fig. 6A to C). In addition, the introduction of a plasmid harboring an intact *covRS* operon into the LMG18311 Δ*covRS* strain rescued the loss of P*_comX_* repression (Fig. 6B). Notably, the impact of CovRS inactivation was much more pronounced in the LMG18311 background than in the spontaneously transformable LMD-9 strain (Fig. 6B and C), suggesting different levels of CovRS repression between the two strains. By comparing *comR* promoter sequences, we observed a high sequence conservation and a similar distribution of ATTARA motifs between the two strains (Fig. S4A). We then aligned CovR and CovS sequences from representative strains of the salivarius group and found a specific mutation (D98E) at a conserved position in CovR-LMD-9 compared to CovR-LMG18311 (Fig. S4B). To investigate the role of this mutation in spontaneous competence activation of strain LMD-9, we exchanged the *covR*S operon and the point mutation between the two strains. As shown in Fig. 6B, the reciprocal exchange of CovRS or D98E mutation alone inverted the level of P*_comX_* activation between the two genetic backgrounds. Notably, due to the D98E mutation, the *comX* expression in strain LMG18311 increased to a sufficient level to unleash natural transformation. The measured transformation rate of various clones was ∼10^−4^, which is similar to strain LMD-9 (40). These results show an altered functionality of CovR_D98E_ that is responsible for a spontaneous transformation in strain LMD-9.

10.1128/mBio.03125-21.4 FIG S4 Conservation of P*_comR_* and CovR in salivarius streptococci. (A) Alignment of P*_comR_.* Asterisks indicate consensus nucleotides. The putative extended −10 box (TNTGNTATAAT) is indicated in purple. ATTARA sequences are highlighted by colored boxes (green, perfect match; blue, one mismatch with TT conservation, gray, two mismatches with ATT conservation). (B) Alignment of CovR performed with the aid of the PRALINE multiple alignment tool (http://www.ibi.vu.nl/programs/pralinewww). Asterisks denote consensus residues. Four representative strains were used for both alignments: *S. salivarius* HSISS4, S. thermophilus LMD-9, S. thermophilus LMG18311, and *S. vestibularis* NTC12167. Download FIG S4, PDF file, 0.9 MB. Copyright © 2022 Knoops et al. 2022 Knoops et al. https://creativecommons.org/licenses/by/4.0/ This content is distributed under the terms of the Creative Commons Attribution 4.0 International license.

Finally, as CovR proteins and *comR* promoters of *S. salivarius*, S. thermophilus, and Streptococcus vestibularis show a high level of conservation (Fig. S4), we performed EMSAs with the purified CovR_HSISS4_ and the different promoter sequences. We observed a significant shift of the three promoter probes that validated direct interactions between partners (Fig. 6D and E).

Together, those data show that CovRS-mediated competence repression is a common feature in the salivarius group of streptococci. Moreover, a point mutation that partially abrogates CovR functionality could explain the spontaneous competence behavior of some S. thermophilus strains under laboratory conditions.

### CovR controls bimodal competence activation in the salivarius group.

CovR is a major repressor of *comR* expression, and we subsequently examined whether we could generate bimodal competence activation through CovRS fine-tuning. For this purpose, we set up a conditional mutation system based on a clustered regularly interspaced short palindromic repeats interference (CRISPRi) module targeting *covRS* expression. Using an optimized LacI repressor together with a dead Cas9 (dCas9) under the control of a P*_lac_* promoter (41), we first validated the system in *S. salivarius* (Fig. S5A and B) and used it to repress the transcription of the *covRS* operon using a guide RNA (gRNA) targeting the *covR* promoter (Fig. S5C). The conditional repression of *covRS* increased P*_comR_* and P*_comX_* activities to a sufficient level to trigger competence (Fig. S5D). However, a strong growth defect associated with abnormal cell morphology and cell aggregation was also observed (Fig. S5E and F). Although we were able to show bimodal competence activation through CovR depletion in *S. salivarius* (Fig. S5F), determining whether toxicity contributes to heterogeneity remained challenging.

10.1128/mBio.03125-21.5 FIG S5 CovR depletion is toxic in *S. salivarius*. (A) Strategy used to validate the CRISPR interference (CRISPRi) system by targeting a constitutive promoter (P_32_) (strategy used in panel B). Together with the reporter system (P_32_*-luxAB*), the engineered strain carries an optimized *lacI* repressor under the control of a constitutive promoter (P_F6_*-lacI*), a nuclease mutant of Cas9 (dCas9) under the control of a lactose-inducible promoter (P*_lac_-dcas9*), and a guide RNA targeting the P_32_ promoter under the control of a constitutive promoter (P_3_*-gRNA_3*). (B) Fold change (percentage) of specific luciferase activity (RLU/OD_600_) for increasing concentration of IPTG in a constitutively expressed luciferase strain (P_32_*-luxAB*). Points show the values for biological triplicates (*n *= 3), while the green bar shows the mean. (C) CRISPRi strategy for *covRS* repression (strategy used in panels D to F). (D) CovR depletion increase *comR* and *comX* transcription. A strain harboring the *dcas9* module reported in panel C together with no guide RNA (WT) or with a guide targeting a single nucleotide modified promoter of *covRS* (*-|covR*) and harboring a P*_comR_-luxAB* or P*_comX_-luxAB* reporter system were incubated with no IPTG or 100 μM IPTG, and maximum specific luciferase activity was recorded. One-way ANOVA with Dunnett’s test was performed for technical triplicates (ns, not significant; *, *P* < 0.05, ****, *P* < 0.0001). (E) Growth defect upon *covRS* inhibition. The strain harboring the *dcas9* module with (*-|covR*) or without (WT) a guide targeting P*_covRS_* reported in panel C were incubated in the presence of 0, 10, or 100 μM IPTG. Solid lines represent average measurements, and shaded lines represent the standard errors. (F) Microscopic single-cell *covRS* repression. The same constructs as described in panel C were used, except that the reporter system was P*_comX_-gfp^+^.* The first vertical set of pictures show the WT, and the second and third sets show the *covRS*-repressed strain with 100 μM IPTG. The second and third vertical sets highlight heterogeneous *comX* activation and aggregation phenotype, respectively. Download FIG S5, PDF file, 2.5 MB. Copyright © 2022 Knoops et al. 2022 Knoops et al. https://creativecommons.org/licenses/by/4.0/ This content is distributed under the terms of the Creative Commons Attribution 4.0 International license.

**FIG 7:**
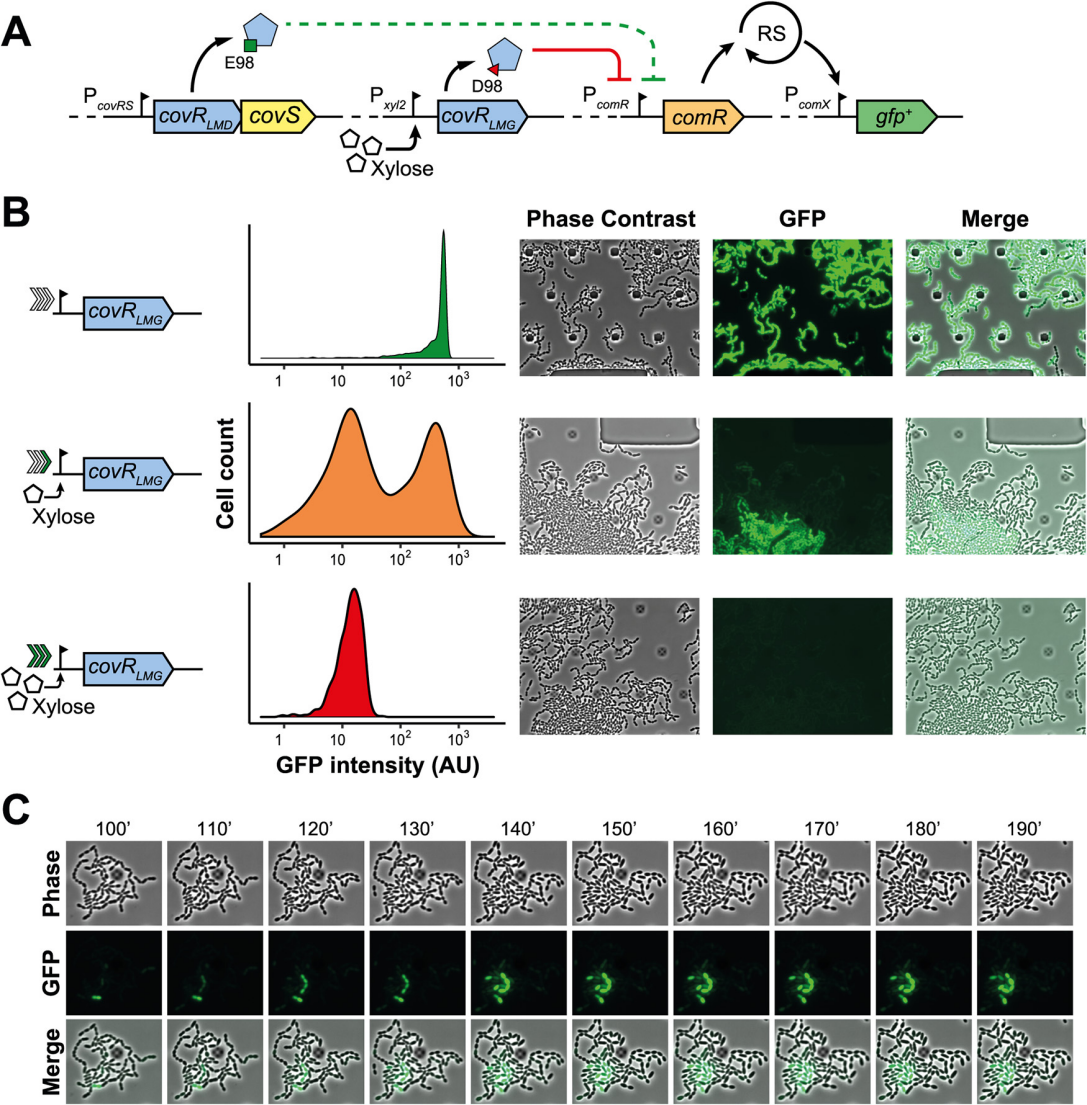
CovR controls competence bimodality. (A) Schematic view of the merodiploid LMD-9 strain (*covR*_LMD_, CovR_E98_) expressing the xylose-inducible wild-type *covR* allele from the LMG18311strain (*covR*_LMG_, CovR_D98_) used in panels B and C. RS, signal amplification through ComRS. (B) Gradual overexpression of *covR*_LMG_ fused to P*_xyl2_* in LMD-9 strain harboring the *covR*_LMD_ mutated gene. The strain was grown for 4 h in M17G, washed, and incubated for 1 h in CDMG prior to loading into a microfluidic device fed with CDMG. Cells with no/mild/high induction (increasing green arrows) were preincubated with 0/0.5/0.5% xylose in M17G, 0/0.5/0.5% xylose in CDMG, and with 0/0/0.5% xylose in the microfluidic device fed with CDMG, respectively. Density plots show the distribution (*n *> 600) of fluorescence, and representative pictures are displayed on the right side, taken after 150 min of growth. (C) Time lapse showing bimodal activation of competence with a *covR*_LMG_ mild-induced condition. Time is shown in minutes (′).

Taking the problem the other way around, we investigated whether we could turn a unimodal activated system due to a lack of CovR functionality, as expected in S. thermophilus LMD-9 (CovR_E98_, *covR*_LMD_), into a bimodal system by overexpressing *covR* from strain LMG18311 (CovR_D98_, *covR*_LMG_). To this aim, we inserted the *covR*_LMG_ allele under the control of P*_xyl2_* (P*_xyl2_-covR*_LMG_) at an ectopic locus together with a P*_comX_-gfp*^+^ reporter fusion in the LMD-9 background (Fig. 7A). Microscopic observations in a microfluidic device of the merodiploid strain showed 100% of P*_comX_* activation in the noninduced cell population and no activation with the fully xylose-induced *covR*_LMG_ gene (Fig. 7B). Interestingly, we were able to produce a bimodal activation by fully inducing *covR*_LMG_ in a preculture, which was further cultivated in a xylose-free medium to generate CovR_LMG_ depletion by cell division and subsequent dilution (Fig. 7B). Time-lapse experiments performed in the same condition illustrate heterogeneous initiation of competence (Fig. 7C).

Altogether, these results strongly suggest that controlling CovRS activity allows to reproduce the effect of ComR on the bimodal activation of competence in salivarius streptococci.

## DISCUSSION

Investigating how environmental sensors influence individual cell fates and elicit bet-hedging strategies in the context of competence is of prime importance to understand how bacteria face different stresses collectively. In the present study, we selected *S. salivarius* as a representative species of ComRS-containing streptococci based on the presence of a single competence signaling system (i.e., ComRS) and absence of spontaneous transformation under laboratory conditions. Our results showed that intracellular levels of the transcriptional sensor ComR and noise amplification through the production of the pheromone ComS/XIP (feedback loop) are the key factors determining the bimodal activation of natural competence in *S. salivarius*. Notably, we revealed the direct transcriptional repression of *comR* by the CovRS system, a TCS controlling virulence in pathogenic streptococci and reported here for the first time as a key player in competence regulation.

**FIG 8:**
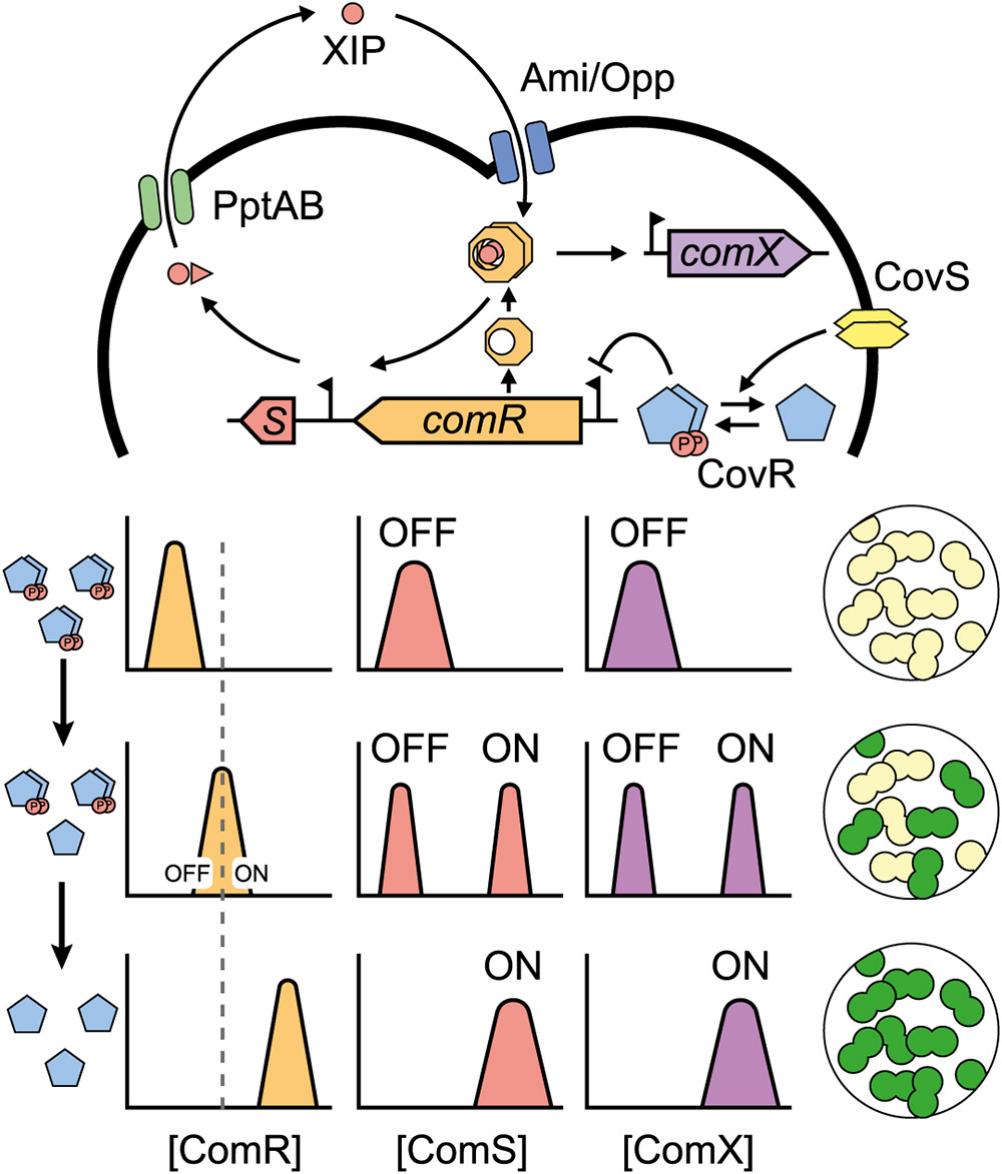
Model for competence bimodal activation in salivarius streptococci. The environmental sensor CovS interacts with CovR through its phosphatase/kinase activity to tune the phosphorylation state of CovR at position D53. Phosphorylated CovR will dimerize and bind P*_comR_* to repress *comR* transcription, while unphosphorylated CovR will not. Low levels of phosphorylated CovR will trigger an increase of ComR concentration to a level sufficient to launch the positive-feedback loop driven by *comS* (S). The loop will be activated in part of the population generating bimodality either because an increase of ComR concentration (i) will lower the ComS threshold necessary for activation and noise of ComS concentration will be amplified or (ii) will shift ComR distribution and noise of ComR concentration will be amplified. Both scenarios will lead to the emergence of two subpopulations (on/off) for *comX* and *comS* activation, but *comR* expression will remain unimodal.

Altogether, those data suggest a new model for the salivarius group where CovRS fine-tunes the amount of ComR which plays a pivotal role in bimodal competence activation (Fig. 8). Particularly, our results suggest two nonexclusive mechanisms that could be at work to explain bimodality in ComRS streptococci: an increase of ComR that would result in the following: (i) a Gaussian distribution of ComR levels, with a subfraction of the population producing enough ComR to exceed the critical concentration threshold that activates competence; and (ii) a decrease of the ComS threshold necessary for nonlinear activation, ultimately generating bimodality from ComS noisy concentration amplification. These mechanisms might be extended to other species and explain why bimodality can be observed only in complex media upon BlpRH activation in S. mutans. Indeed, transcriptomics data indicated that BlpRH is able to upregulate ComR only in complex media in this species (10, 14). Those results suggest that, similarly to the salivarius group, an increase of ComR concentration could account for bimodality. Besides, the control of ComR levels by CovRS in *S. salivarius* mirrors the control of ComRS by BlpRH in S. mutans. The first one is known as an environmental sensor, while the second is a pheromone communication device. The use of differing distal regulators in both species is probably linked to their distinct lifestyles and specific needs to react toward different competence stimuli.

In addition to a dissimilar distal control of ComRS, the pheromone-based induction system of competence behaves differently between *S. salivarius* and S. mutans. In this work, we have shown the necessity for a pheromone exporter (PptAB) and a peptide importer (Ami/Opp) for competence activation through ComR overproduction (Fig. 3). This suggests that the pheromone ComS must be exported, maturated, and reimported in order to generate a functional positive-feedback loop in *S. salivarius*. Conversely, PptAB does not seem to be essential for ComS export in S. mutans (42), and its Opp system was shown to be dispensable for bimodal activation, presumably because of nonmaturated ComS intracellular activity (11, 43). Furthermore, the pheromone overproduction does not trigger competence in *S. salivarius* (Fig. 1), in contrast with the S. mutans model (43). Those data underline the specific low sensitivity of the ComRS system toward subtle-to-mild ComS increase at native ComR levels in *S. salivarius*. This suggests that this species has evolved a negative player locking the entry into competence through ComS limitation, which is counterintuitive regarding the fact that ComRS is sensitive to the addition of low exogenous sXIP concentrations (Fig. 2D). A negative player such as a pheromone-specific peptidase described in other streptococci could explain this discrepancy (44, 45). While peptidase processive activity could impair the progressive intrinsic peptide production, exogenous sXIP addition would rapidly saturate the degradation apparatus.

To our knowledge, the inhibition of competence through CovRS has never been reported in streptococci. While transcriptomics data suggest a positive correlation between the standalone CovR and competence activation in S. mutans (46), no link between CovRS and competence was reported in GAS (group A streptococci) or GBS (group B streptococci), despite extensive transcriptomics analyses (29, 33, 47–49). Notably, we show that a natural mutation altering CovR function (i.e., CovR_D98E_) is associated with spontaneous transformation in salivarius streptococci while CovRS mutations generate hypervirulent strains in GAS (50). Analogously, natural mutations in competence repression systems of bacilli were previously reported to explain spontaneous transformation of specific isolates under laboratory conditions (51). Our mutagenesis results obtained with the nonphosphorylatable (D53A) and the phosphomimetic (D53E) alleles of CovR are in accordance with previous reports of GAS and GBS, showing an increase of transcriptional repression for the phosphorylated form of CovR (38, 52). As expected, the phosphatase mutant (T287A) of CovS showed a decrease in *comR* expression since more abundant CovR∼P is predicted. Whether CovS in GAS has mostly a phosphatase or kinase activity is still unclear (34, 53–55). In *S. salivarius*, we showed that a Δ*covS* mutant displays a strong *comR* repression (Fig. 4A), suggesting a high CovR∼P level and consequently the existence of alternative phosphorylation mechanism(s) (e.g., TCS cross talk or small phospho-donor molecules) as hypothesized for GAS and GBS (34, 56). While this implies that CovS has mostly a phosphatase role in our experimental conditions, the environmental trigger(s) of this activity remains challenging to discover. In GAS and GBS, the CovS triggering stimuli have been extensively studied. While CovS of GAS reacts to pH, human defensin LL-37, osmotic shock, amino acid starvation, temperature, and iron starvation (31, 33, 57, 58), the GBS CovS has been reported to react only to pH and glucose availability (59, 60). Environmental trigger identification performed in this work confirmed previous data (39), suggesting a pH dependency of the CovRS activity in the salivarius group (Fig. S6). However, we were unable to generate a response for the stimuli reported in GAS (Fig. S6). Because competence activation is extremely species specific and depends on the bacterial lifestyle (3), it is not surprising to find that commensals belonging to the salivarius group do not respond to the same stimuli as GAS/GBS pathogens.

10.1128/mBio.03125-21.6 FIG S6 CovRS triggering conditions. (A) Investigations of putative environmental triggers of P*_comR_* activation by CovR. We compared the P*_comR_-luxAB* reporter strain (WT) and the mutant strain impaired for its CovRS pathway (*covR*_D53A_, P*_comR_-luxAB*). Fold changes for each condition were calculated by dividing values of maximum specific luciferase activity (RLU/OD_600_) obtained for stimulus induction with noninduced mock values (BCAA, branched chained amino acid). For details on stimulus features, see Materials and Methods. (B) Influence of pH on P*_comR_* activation by CovR. Maximum specific luciferase activity (RLU/OD_600_) of P*_comR_-luxAB* fusion in WT compared to the *covR*_D53A_ mutant at various initial pHs (buffered CDM). Mean values of technical triplicates ± standard deviations are shown. In panel A, a statistical *t* test was performed for each condition compared to their relative mock values (*, *P* < 0.05). Download FIG S6, PDF file, 0.5 MB. Copyright © 2022 Knoops et al. 2022 Knoops et al. https://creativecommons.org/licenses/by/4.0/ This content is distributed under the terms of the Creative Commons Attribution 4.0 International license.

This study of the molecular mechanisms hidden behind competence bimodal activation in *S. salivarius* helps us to understand how bacteria can integrate external stimuli and translate them into a quantitative subpopulation activation. This collective survival strategy is of major importance for subsisting in highly competitive environments such as the human digestive tract, and studying it broadens our understanding of its dynamics in bacterial communities.

## MATERIALS AND METHODS

### Bacterial strains, plasmids, and oligonucleotides.

Bacterial strains, plasmids, and oligonucleotides used in this study are listed and described in the supplemental material (see Tables S3A to S3C in the supplemental material).

10.1128/mBio.03125-21.9 TABLE S3 List of bacterial strains (A), plasmids (B), and oligonucleotides (C) used in this study. Download Table S3, PDF file, 0.3 MB. Copyright © 2022 Knoops et al. 2022 Knoops et al. https://creativecommons.org/licenses/by/4.0/ This content is distributed under the terms of the Creative Commons Attribution 4.0 International license.

### Growth conditions.

S. salivarius HSISS4 (61), S. thermophilus LMG 18311, and S. thermophilus LMD-9 derivatives were grown at 37°C without shaking in M17 (Difco Laboratories, Detroit, MI) or in chemically defined medium (CDM) (62) supplemented with 1% (wt/vol) glucose (M17G or CDMG, respectively). Escherichia coli TOP10 (Invitrogen) was cultivated with shaking at 37°C in LB (lysogeny broth). Chromosomal genetic constructions were introduced in *S. salivarius* and S. thermophilus via natural transformation (40). Electrotransformation of E. coli was performed as previously described (63). We added d-xylose (0.1% to 1% [wt/vol]), isopropyl-β-d-thiogalactopyranoside (IPTG) (1 mM), ampicillin (250 μg/ml), spectinomycin (200 μg/ml), chloramphenicol (5 μg/ml), erythromycin (10 μg/ml), and kanamycin (500 μg/ml) as required. The synthetic peptide XIP (sXIP, LPYFAGCL, purity of 95%), was supplied by Peptide 2.0 Inc. (Chantilly, VA) and resuspended in water. Solid plates inoculated with streptococcal cells were incubated anaerobically (BBL GasPak systems, Becton Dickinson, Franklin Lakes, NJ) at 37°C. When stated, the peptide LL-37 supplied by Proteogenix (Schiltigheim, France) (1 μM, purity of 95%), NaCl (300 mM), sorbitol (7% vol/vol), H_2_O_2_ (0.01% vol/vol), or branched-chain amino acids (valine, leucine, and isoleucine at final concentrations of 7.5 mM, 9.7 mM and 4.3 mM, respectively) were added as supplements to CDMG. A metal depletion solution was prepared by adding all the CDM components except MgCl_2_ · (H_2_O)_6_, CaCl_2_ · (H_2_O)_4_ and Mn(II)SO_4_ · H_2_O.

### Competence induction, donor DNA, and validation of mutants.

To induce competence, overnight CDMG precultures were diluted to a final optical density at 600 nm (OD_600_) of 0.05 in 500 μl of fresh CDMG and incubated at 37°C for 100 min. Then, the pheromone sXIP and DNA (overlapping PCR products or plasmids) were added, and the cells were incubated for 3 h at 37°C before plating on M17G agar supplemented with antibiotics when required. All the details used to generate overlapping PCR for the construction of mutants reported below are available in Table S4 in the supplemental material. The integrity of all the constructed mutants was validated by Sanger DNA sequencing.

10.1128/mBio.03125-21.10 TABLE S4 List of (overlapping) PCR fragments, synthetic DNA constructs, and EMSA probes. Download Table S4, PDF file, 0.2 MB. Copyright © 2022 Knoops et al. 2022 Knoops et al. https://creativecommons.org/licenses/by/4.0/ This content is distributed under the terms of the Creative Commons Attribution 4.0 International license.

### Construction of deletion mutants.

Null mutants were constructed by exchanging (double homologous recombination) the coding sequences (CDS) of target genes (sequence between start and stop codons) for either chloramphenicol or erythromycin resistance cassette (*cat* or *erm*). If stated, the *lox* site flanked resistance cassette was excised from mutants using the Cre-*lox* system as previously described (40). Integration of the antibiotic resistance cassette at the right location was subsequently checked by PCR.

### Construction of overexpression mutants and reporter systems.

Gene overexpression systems were designed by ATG fusion either to a mild xylose-inducible promoter (P*_xyl2_*, associated with the *xylR* regulator gene), to a strong constitutive promoter (P_32_) or to a strongly locked Streptococcus pneumoniae improved version of an IPTG-inducible promoter (P*_lac_*, associated with a P*_F6_-lacI* repressor [41]). Those constructs were linked to a *cat* cassette and integrated at the permissive tRNA serine locus (*HSISS4_r00062* for HSISS4 and *STER_t1568* for LMD-9) or fused to the native gene by double homologous recombination. To overexpress *comS* or *xip* fused to P*_xyl2_* associated with a *cat* cassette, we used the permissive locus *tnpII* (downstream of *HSISS4_01854*).

The competence promoters were fused to the *luxAB* or *gfp^+^* reporter gene and inserted with a spectinomycin resistance cassette (*spc*) at the permissive tRNA threonine locus (*HSISS4_r00061*) for strain HSISS4, at the *blp* locus (*stu_1673*) for strain LMG18311, or at the *suc* locus (*ster_1709*) for strain LMD-9 or directly downstream of *comR* by double homologous recombination. For green fluorescent protein (GFP) constructs P*_comX_-gfp^+^*, P*_xyl2_-gfp^+^*, and P*_comR_-gfp^+^*, a codon-optimized sequence for GFP was specifically designed for use with an optimized 5′-UTR sequence, in order to maximize translation efficiency and improve GFP expression (designed by MeKaGene, Belgium). Synthetic constructs were ordered as Gblocks at IDT (Leuven, Belgium). They were then joined to an antibiotic resistance cassette and homologous chromosomal regions via overlapping PCR. For CovR mutants, residue mutations in the protein sequence were introduced with nucleotide mismatch primers. A *cat* cassette and homologous genomic regions for double recombination were associated with the construction by overlapping PCR.

### Construction of CRISPRi conditional mutants.

For dCas9-mediated (Cas9 catalytic mutant) transcriptional inhibition of *covRS* and P_32_-*luxAB* (validation tool), *lacI* was amplified from plasmid pPEPY-P*_F6_*-*lacI* (41), Gibson assembled to HSISS4 recombination arms together with a *spc* cassette and inserted at the tRNA threonine locus by natural transformation. The same technique was used to transfer *dcas9* under the control of a P*_lac_* promoter (amplified from JWV102-P*_lac_*-*dcas9*_sp_ [41]) with a *cat* cassette into the tRNA serine locus. The two resistance cassettes were concomitantly excised as previously described (40), and a P*_comX_*-*luxAB* or P_32_-*luxAB* reporter system fused to *spc* were introduced into the *tnpII* locus. For *covR* inhibition, a P*_xyl2_-comR-cat* construct was introduced at the *suc* locus (upstream of *HSISS4_01641*), and both resistance cassettes at the *tnpII* and *suc* loci were excised. Finally, a constitutive promoter (P_3_) and a *dcas9* handle followed by an S. pyogenes terminator were amplified from plasmid pPEPX-P_3_-sgRNAluc (41), fused to a specific gRNA targeting P*_covR_*, and introduced with an *erm* cassette into a fifth permissive locus (*gor*, downstream of *HSISS4_00325*). Since P*_covR_* has no protospacer adjacent motif (PAM) sequences, we introduced a point mutation into the promoter (spacer between −35 and −10 boxes) to generate the NGG sequence necessary for dCas9 binding.

### Luciferase activity.

Overnight precultures were diluted to a final OD_600_ of 0.05. A volume of 300 μl of culture was transferred into the wells of a sterile covered white microplate with a transparent bottom (Greiner, Alphen a/d Rijn, The Netherlands). These culture samples were supplemented with d-xylose, IPTG, LL-37, NaCl, sorbitol, H_2_O_2_, or branched-chain amino acids (BCAA) if stated or further incubated for 100 min at 37°C before supplementation with sXIP (1 μM). Growth (OD_600_) and luciferase (Lux) activity (expressed in relative light units [RLU]) were monitored at 10-min intervals for 8 h in a Hidex Sense microplate reader (Hidex, Lemminkäisenkatu, Finland). Maximum specific Lux activity was obtained by dividing Lux activity by the OD_600_ over time and selecting the maximum value obtained. Biological or technical triplicates were then averaged. Statistical analyses of simple and multiple comparisons to the control mean were performed with *t* test (unilateral distribution, heteroscedastic) and one-way analysis of variance (ANOVA) with Dunnett’s test, respectively. For both, standard deviations and *P* values were calculated.

### CovR and ComR purification.

First, the PCR-amplified *covR*_WT_, *covR*_D53A_, and *covR*_D53E_ genes (from strains HSISS4, AK0011, and AK0012, respectively) were cloned into the pBAD-comR-ST vector (22). Because the DNA-binding domain of CovR is predicted at the C terminus, the StrepTag was placed at the N terminus by Gibson assembly, and the final construct was verified by DNA sequencing. The ComR-StrepTag or StrepTag-CovR recombinant proteins were overproduced in E. coli and purified in standard native conditions on Strep-Tactin agarose beads (IBA, Germany) as previously described (18).

### Electrophoretic mobility shift assays (EMSAs).

All double-stranded DNA fragments (150 bp) were obtained by PCR with genomic or synthetic DNA as the template. PCR was performed with either Cy3-labeled (at the 5′ end) and unlabeled oligonucleotides or forward Cy3-labeled and reverse Cy5-labeled primers for double fluorescence. Primers used are listed in Table S4. Typically, a gel shift reaction (20 μl) was performed in a binding buffer [20 mM Tris, 1 mM CaCl_2_, 1 mM dithiothreitol (DTT), 10 μg/ml poly(dI-dC), and 100 μg/ml bovine serum albumin (BSA), pH 7.5] (36) and contained 100 ng of labeled probe and 1.4 μM with 1.4 × serial dilutions of purified Strep-tagged CovR_WT/D53A/D53E_. The reaction mixture was incubated at 37°C for 5 min without the probe to get rid of any unspecific binding and for 10 min after adding the probe prior to the loading of the samples onto a native 4 to 20% gradient gel (iD polyacrylamice gel electrophoresis [PAGE] gel; Eurogentec, Belgium). The gel was run at 100 V for 100 min in MOPS buffer (50 mM Tris-base [pH 7.7], 50 mM morpholinepropanesulfonic acid [MOPS], 1 mM EDTA). DNA complexes were detected by fluorescence on an Ettan DIGE Imager (GE Healthcare, Waukesha, WI) with bandpass excitation filters of 540/25 nm (Cy3) or 635/30 nm (Cy5) and bandpass emission filters of 595/25 nm (Cy3) or nm 680/30 (Cy5). Gel fluorescence intensity quantification, background subtraction and normalization were performed with ImageQuant array (GE Healthcare). Those quantified intensities were then used to compute the percentage of bound probes with the formula PB=100−PU−bctrl×100 where *P_U_* stands for unbound probe, *b* stands for background, and ctrl stands for control (for the probe without CovR addition).

For experiments with restriction fragments of the *comR* promoter, P*_comR_* was PCR amplified using primers AK304-CY3 and AK326-CY5 and purified using ethanol extraction. The fragment was then digested with NsiI, PsiI, DraI, AseI, or PsiI-NsiI and purified overnight by ethanol extraction. One hundred nanograms of digested DNA was used for the experiment as previously described.

For the binding assay of CovR_WT_ to P*_comR_* from various streptococci, synthetic DNAs corresponding to P*_comR_* (150 bp) of three salivarius streptococci (*S. salivarius* HSISS4, S. thermophilus LMD-9, and *S. vestibularis* NTC1267) and one negative control (P*_dnaE_* from HSISS4) were ordered at IDT (Leuven, Belgium). Flanking sequences of 20 bp were chosen arbitrarily to amplify all the sequences with primers AK354-CY3 and AK355. Statistical analyses were performed as reported for luminescence assays.

### Immunoblotting.

Protein samples were separated by sodium dodecyl sulfate-polyacrylamide gel electrophoresis (SDS-PAGE) on 12% acrylamide precast gel (iD PAGE Gel; Eurogentec) and washed in Tris-glycine-SDS solution (0.6% [wt/vol] Tris-HCl, 0.3% [wt/vol] glycine, 0.02% [wt/vol] SDS in 8:2 water-methanol solution) for 30 min. Gels were then blotted onto 0.2-μm nitrocellulose membrane with TransBlot Turbo transfer system (Bio-Rad) and subsequently blocked for 1 h with Tris-buffered saline (20 mM Tris base; 150 mM NaCl) containing 0.05% (vol/vol) Tween 20 (TBST) and 5% (wt/vol) dry milk (TBST-milk). The membranes were then incubated for an additional hour with the primary antibodies diluted in TBST-milk. The membranes were washed four times (5 min each) in TBST and incubated for 1 h with the secondary antibody diluted in TBST-milk. The membranes were finally washed again four times (5 min each) in TBST, revealed with Immobilon Western blotting chemoluminescence HRP substrate (Merck Millipore), and visualized with an Amersham Imager 600 (GE Healthcare). Rabbit antisera against ComR (anti-ComR) were used at a 1:5,000 dilution. Horseradish peroxidase (HRP)-conjugated anti-rabbit secondary antibody was used at a 1:20,000 dilution (Jackson ImmunoResearch, West Grove, PA). ComR amount was quantified and normalized using total protein stained with Coomassie blue using an Amersham Imager 600 (GE Healthcare).

### Microscopy.

CDMG overnight precultures were diluted in fresh CDMG at an OD_600_ of 0.05 and incubated at 37°C for 4 h. For CRISPRi depletion of CovRS, cells were incubated under the same conditions but were diluted at an OD_600_ of 0.01 and supplemented with 1 mM IPTG. Cells were then centrifuged, resuspended in fresh CDMG at an OD_600_ of 0.05 with d-xylose, and incubated for 2 h at 37°C. When stated, sXIP was added after 30 min of incubation. Cells were then centrifuged, resuspended in 50 μl of phosphate-buffered saline (PBS), and observed on agarose pads composed of 1% agarose and PBS buffer. For LMD-9 competence bimodality observation, competence nonpermissive M17G medium was used for overnight preculture. Cells were then diluted in fresh M17G at an OD_600_ of 0.05 with or without xylose induction (0.5%) and incubated for 4 h at 37°C. Cells were washed in fresh CDMG, diluted to an OD_600_ of 0.05 in CDMG, and incubated for 1 h at 37°C with or without xylose prior to microfluidic device loading (Cellulasic Onix2; Merck Millipore). The flow pressure was set at 10 kPa, and pictures were taken every 10 min. Images were obtained using an Axio I inverted microscope (Zeiss) equipped with a Plan-Apochromat objective (100 ×/1.46 oil differential interference contrast [DIC] M27) (Zeiss), a HXP 120 C lighting unit (Zeiss), and a C10600 ORCA-R2 camera (Hamamatsu). GFP fluorescence was detected with filter set 38 HE, displaying bandpass excitation 470/40 nm and bandpass emission 525/50 nm (Zeiss). Images were analyzed using ZenPro software (Zeiss) and MicrobeJ (64).

### Deep sequencing (RNA-seq) and data processing.

*S. salivarius* WT, *CovS*_T287A_, and *CovR*_D53E_ strains were precultured overnight in CDMG at 37°C. Precultures were then diluted at an OD_600_ of 0.05 in CDMG, incubated for 4 h at 37°C, and concentrated by centrifugation (2 min, 4,000 × *g*), and OD_600_ was measured. The strains were diluted again at an OD_600_ of 0.2 in 10 ml of CDMG, grown for 25 min, and the supernatants were discarded. The cell pellets were then frozen in liquid nitrogen. RNA was extracted the same day using the RNeasy plus bacteria kit (Qiagen) using the protocol provided by the manufacturer. Total RNA was checked for integrity with a RNA Nano chip (Agilent Technologies). rRNA depletion was performed on 2 μg total RNA with the Ribo-Zero rRNA removal kit for Gram-positive bacteria (Illumina) according to the manufacturer’s instructions. Total stranded mRNA libraries were prepped with the NEBNext Ultra Directional RNA Library Prep kit for Illumina (New England Biolabs). Library PCR was executed for 15 cycles. Quality of the libraries was evaluated with the use of a high-sensitivity DNA chip (Agilent Technologies), and concentrations were determined by quantitative PCR (qPCR) according to Illumina protocol. Libraries were pooled equimolarly for sequencing on a NextSeq 500 high-throughput run for generating 76-bp single reads. 2.3 pM of the library pool was loaded on the flow cell with a Phix spike-in of 5%. Sequenced mRNAs generated several million reads that were mapped on the *S. salivarius* HSISS4 chromosome and processed on the Galaxy server (usegalaxy.org) using the Bowtie2 algorithm to yield BAM files containing the read coordinates and Featurecount to count the number of reads per coding sequence (CDS). The data set was exported into an Excel file for further analyses. First, the data set was standardized to CDS-mapped reads per million overall reads. Then, we estimated a ratio of CDS-mapped reads in mutants versus WT.

### Data availability.

All RNA-seq data were deposited in the GEO database under accession number GSE158512.
