# Supplementary figures and images for: The CovRS Environmental Sensor Directly Controls the ComRS Signaling System To Orchestrate Competence Bimodality in Salivarius Streptococci
Source: mBio. 2022 Jan 4;13(1):e03125-21. doi: 10.1128/mbio.03125-21 (PMC8725580; doi:10.1128/mbio.03125-21)

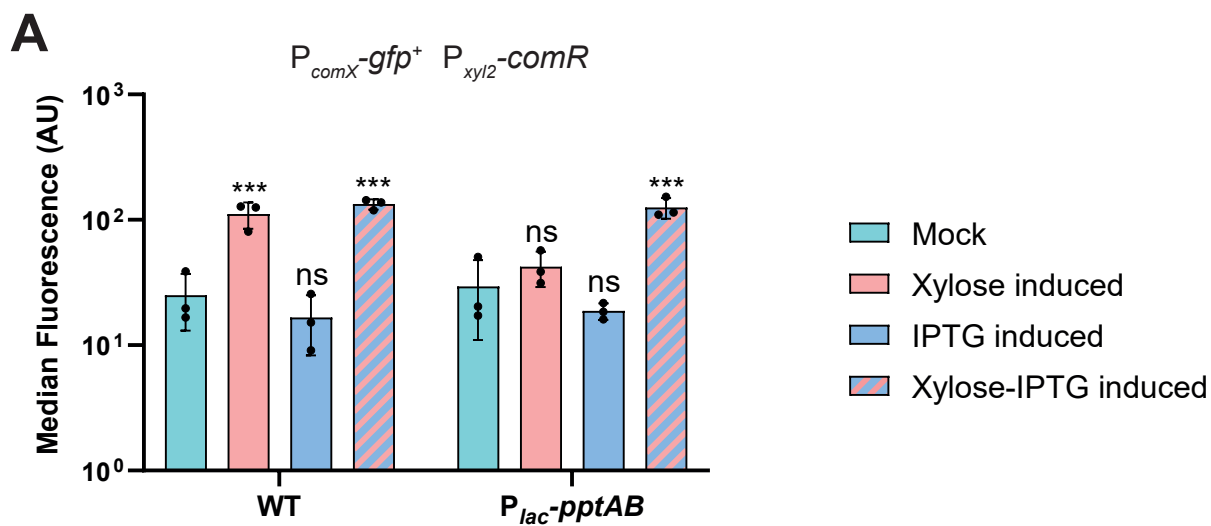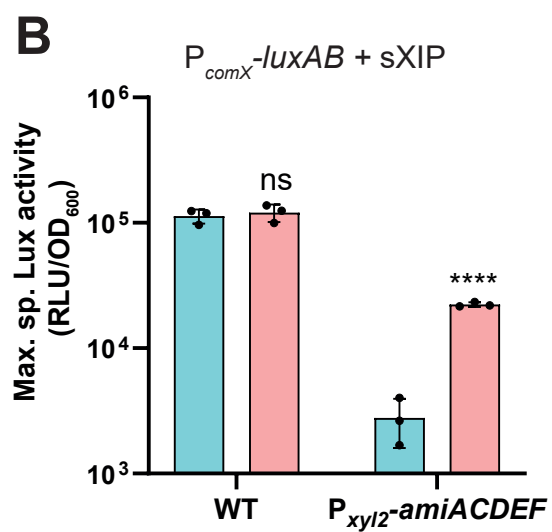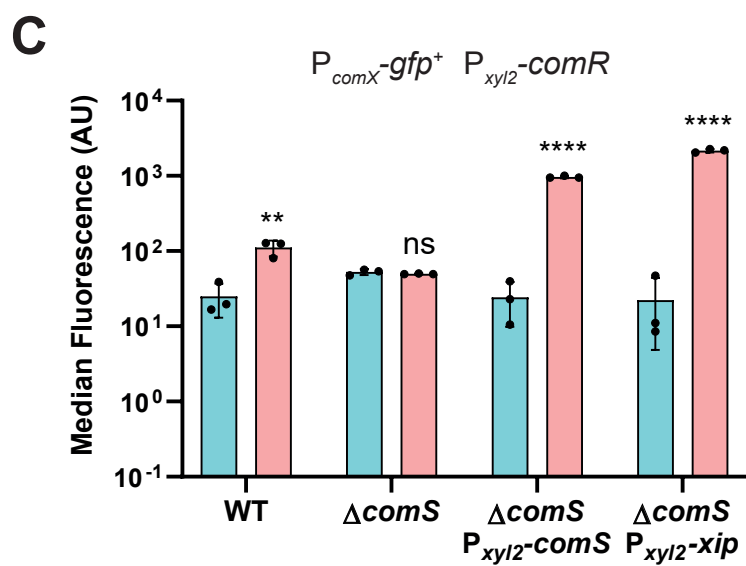

Supplement: FIG S1 [file mbio.03125-21-sf001.pdf]

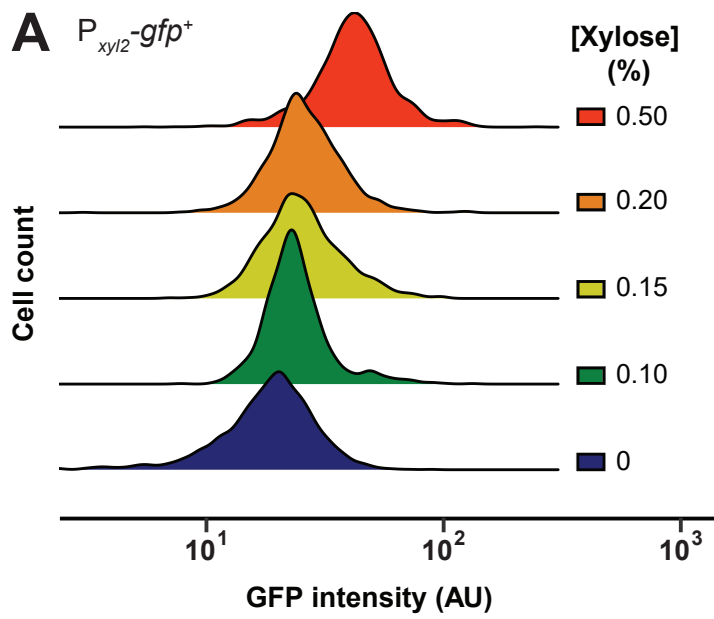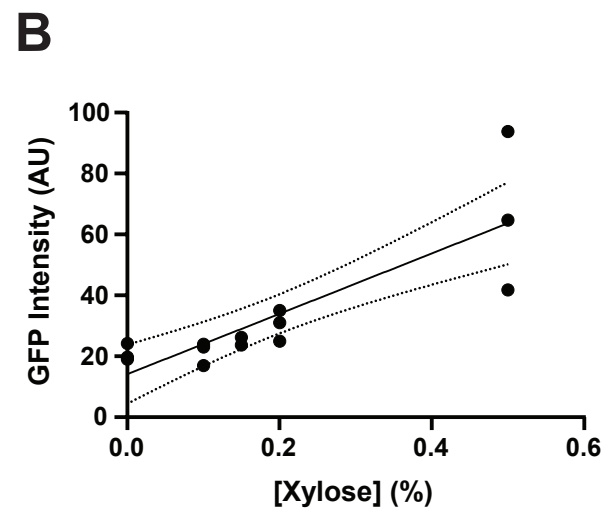

Supplement: FIG S2 [file mbio.03125-21-sf002.pdf]

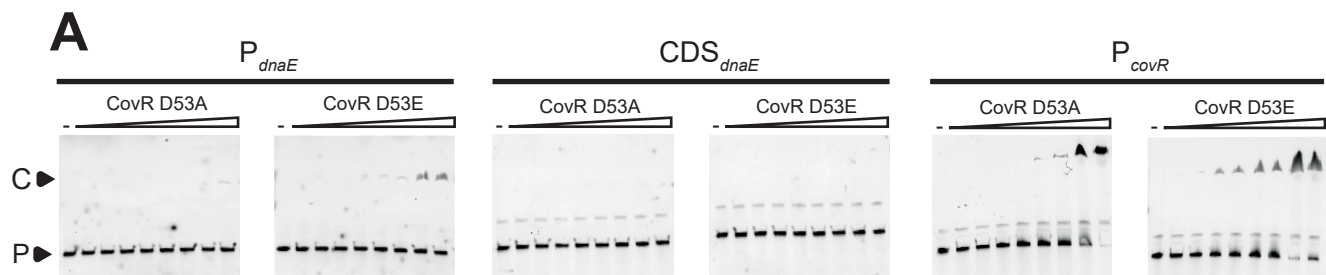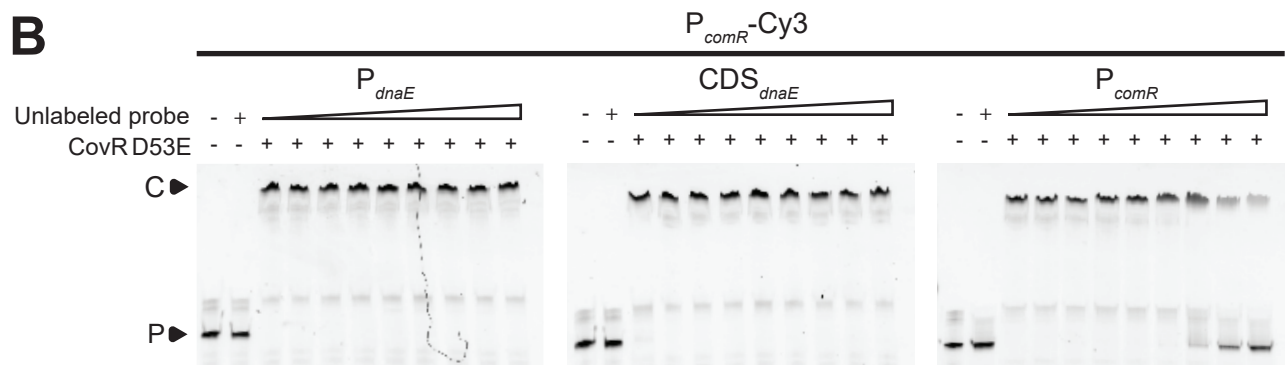

Supplement: FIG S3 [file mbio.03125-21-sf003.pdf]

**A**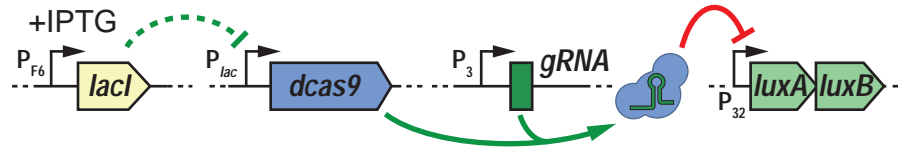**B**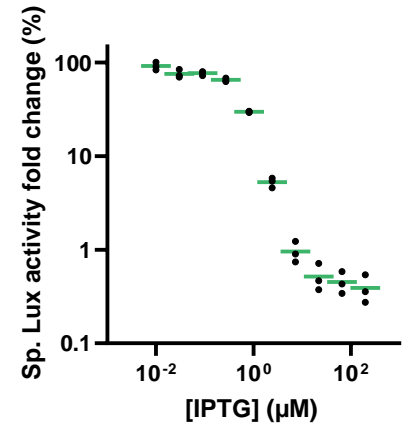**C**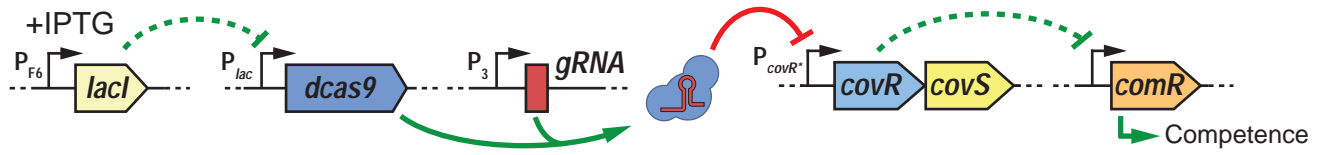**D**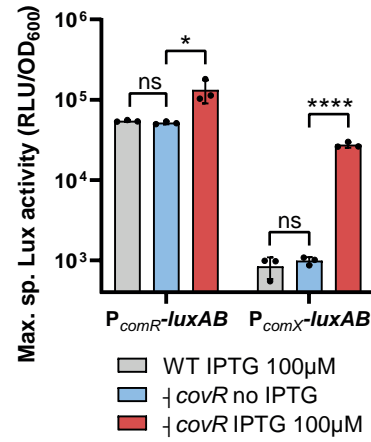**E**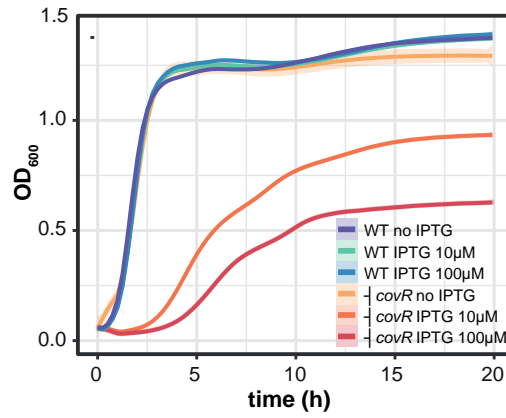**F**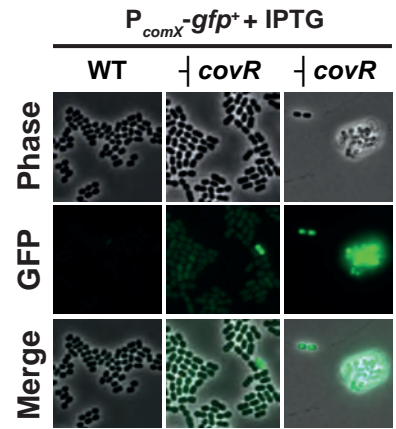

Supplement: FIG S5 [file mbio.03125-21-sf005.pdf]

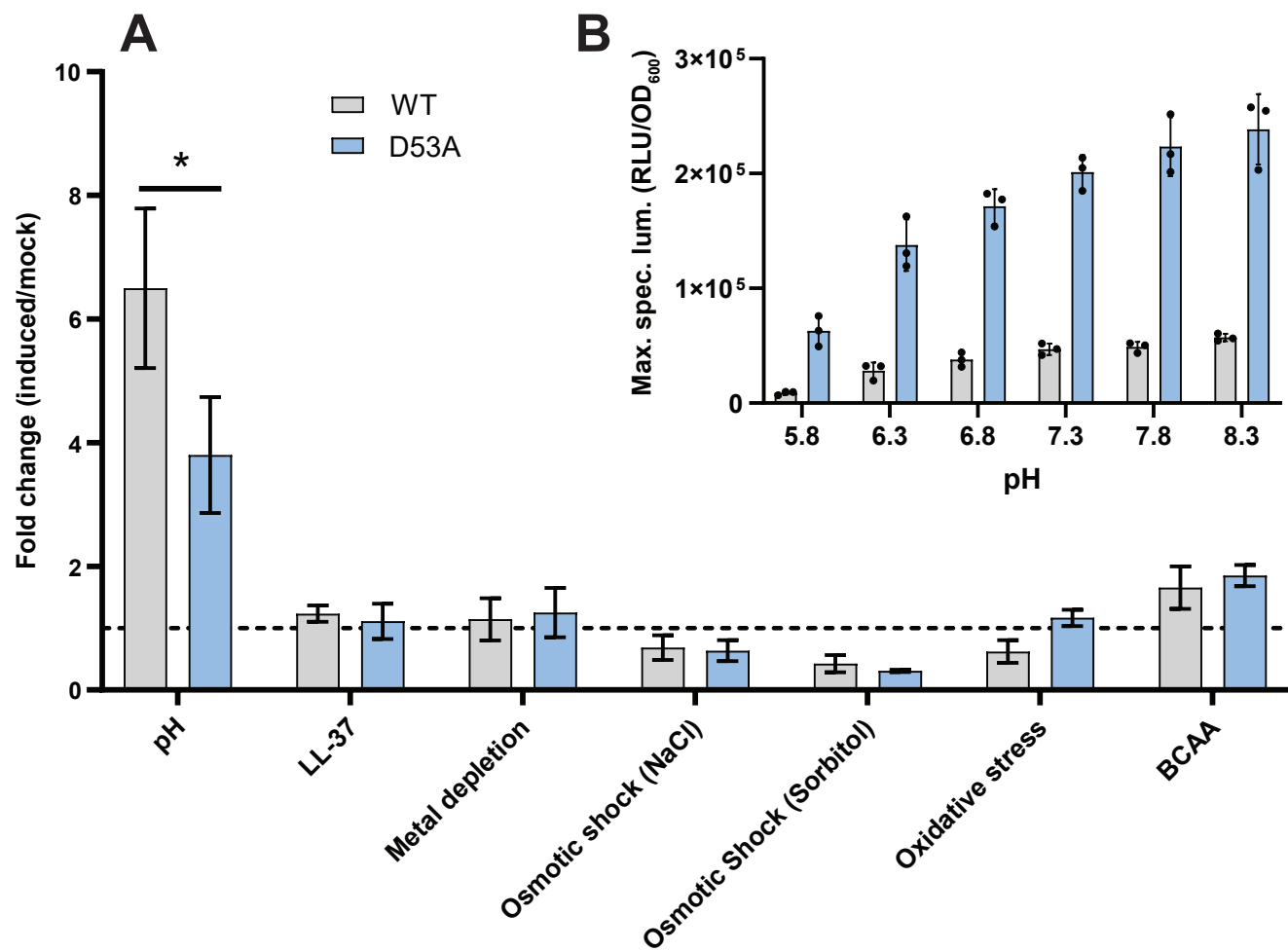

Supplement: FIG S6 [file mbio.03125-21-sf006.pdf]
