# Supplementary material for: The CovRS Environmental Sensor Directly Controls the ComRS Signaling System To Orchestrate Competence Bimodality in Salivarius Streptococci
Source: mBio. 2022 Jan 4;13(1):e03125-21. doi: 10.1128/mbio.03125-21 (PMC8725580; doi:10.1128/mbio.03125-21)
Supplement: TABLE S1 [file mbio.03125-21-st001.pdf]

Table S1. Annotation of TCSs from *S. salivarius* HSISS4

S. salivarius HSISS4 [a]			*S. thermophilus* LMD-9	*S. pneumoniae* R6	*S. pyogenes* M1	*S. mutans* UA159
**Locus_tag**	**Gene name**	**Product**	**Locus_tag / gene name %QC / %Id** [b]	**Locus_tag / gene name %QC / %Id**	**Locus_tag / gene name %QC / %Id**	**Locus_tag / gene name %QC / %Id**
HSISS4_00246	*covR*	RR CovR	STER_RS01725 / *rr01* 100 / 96	SPR_RS01745 / *csrR* 100 / 48	SPY_RS01535 / *csrR* 100 / 82	SMU_RS08745 / *gcrR* 99 / 78
HSISS4_00247	*covS*	HK CovS	STER_RS01730 / *hk01* 96 / 89	ND [d]	SPY_RS01540 / *csrS* 95 / 43	ND
HSISS4_01097	*ciaR*	RR CiaR	*Ψ* [c]	SPR_RS03560 / *ciaR* 99 / 87	SPY_RS05180 / *ciaR* 100 / 84	SMU_RS05200 / *ciaR* 100 / 86
HSISS4_01096	*ciaH*	HK CiaH	*Ψ*	SPR_RS03565 / *ciaH* 99 / 48	SPY_RS05175 / *ciaH* 98 / 52	SMU_RS05195 / *ciaH* 97 / 54
HSISS4_01036	*spaR*	RR	STER_RS04580 / *rr04* 100 / 86	SPR_RS09650 / *pnpR* 99 / 31	SPY_RS04515 / *strR* 99 / 37	SMU_RS03120 / *spaR* 100 / 58
HSISS4_01035	*spaK*	HK	STER_RS04585 / *hk04* 100 / 85	SPR_RS00420 / *pnpS* 65 / 25	SPY_RS04520 / *strK* 73 / 29	SMU_RS03125 / *spaK* 90 / 48
HSISS4_00836	*vicR*	RR vicR	STER_RS05520 / *rr05* 100 / 95	SPR_RS05525 / *vicR* 99 / 80	SPY_RS02235 / *vicR* 100 / 84	SMU_RS06885 / *vicR* 100 / 83
HSISS4_00837	*vicK*	HK vicK	STER_RS05515 / *hk05* 100 / 92	SPR_RS05520 / *vicK* 90 / 72	SPY_RS02240 / *vicK* 93 / 74	SMU_RS06880 / *vicK* 92 / 74
HSISS4_01178	*hk06*	HK	STER_RS06365 / *hk06* 100 / 85	SPR_RS09115 / *hk11* 98 / 35	ND	SMU_RS07025 / *hk12* 99 / 57
HSISS4_01177	*rr06*	RR	STER_RS06360 / *rr06* 100 / 94	SPR_RS09110 / *rr11* 100 / 68	ND	SMU_RS07020 / *rr12* 99 / 74
HSISS4_01193	*rr07*	RR	STER_RS06460 / *rr07* 100 / 98	SPR_RS07330 / *rr01* 100 / 85	ND	SMU_RS04640 / *rr08* 100 / 46
HSISS4_01192	*hk07*	HK	STER_RS06455 / *hk07* 100 / 88	SPR_RS07325 / *hk01* 81 / 65	ND	SMU_RS04645 / *hk08* 79 / 33
HSISS4_01345	*liaS*	HK LiaS	*Ψ*	SPR_RS01780 / *hk03* 91 / 48	SPY_RS06770 / *liaS* 99 / 51	SMU_RS02335 / *hk11* 91 / 57
HSISS4_01344	*liaR*	RR LiaR	STER_RS06830 / *rr08* 100 / 99	SPR_RS01785 / *rr03* 99 / 71	SPY_RS06765 / *liar* 99 / 84	SMU_RS02340 / *rr11* 99 / 84
HSISS4_00378	*rr09*	RR	STER_RS08080 / *rr09* 97 / 36	SPR_RS02375 / *rr13* 99 / 48	ND	SMU_RS08710 / *comE* 99 / 51

HSISS4_00379	*hk09*	HK	STER_RS08085 / *hk09* 95 / 30	SPR_RS02380 / *hk13* 96 / 33	SPY_RS06655 / *hk* 84 / 26	SMU_RS08705 / *comD* 92 / 38
HSISS4_00352	*rr10*	RR	ND	ND	SPY_RS03640 / *sptR* 99 / 60	SMU_RS04280 / *rr04* 100 / 60
HSISS4_00353	*hk10*	HK	ND	ND	SPY_RS03645 / *sptS* 100 / 56	SMU_RS04285 / *hk04* 100 / 53
HSISS4_01248	*vncR*	RR VncR	ND	SPR_RS02675 / *vncR* 100 / 71	SPY_RS08395 / *irr* 99 / 38	ND
HSISS4_01247	*vncS*	HK VncS	ND	SPR_RS02680 / *vncS* 99 / 53	SPY_RS08390 / *ihk* 96 / 26	ND
HSISS4_01715	*fasA*	RR	ND	ND	SPY_RS01155 / *fasA* 99 / 56	ND
HSISS4_01716	*fasB*	HK	ND	ND	SPY_RS01150 / *fasB* 98 / 25	ND
HSISS4_01231	*rr13*	RR	ND	SPR_RS10180 / *rr06* / 87 / 33	ND	SMU_RS04780 / *rr07* 100 / 74
HSISS4_01230	*hk13*	HK	ND	SPR_RS10175 / *hk06* 72 / 25	ND	SMU_RS04775 / *hk07* 91 / 69
HSISS4_01447	*rr14*	RR	ND	SPR_RS10400 / *comE* / 96 / 33	ND	ND
HSISS4_01446	*hk14*	HK	ND	SPR_RS10405 /*comD* / 56 / 31	ND	ND

[a] The TCS number 01 to 09 assigned to *S. salivarius* HSISS4 was based on previous TCS annotation from *S. thermophilus* LMD-9 (1). TCS annotation is based on gene names assigned to orthologous systems in *S. pneumoniae* R6 (2), *S. pyogenes* M1 (3), and *S. mutans* UA159 (4)

[b] %QC, percentage of query covering, %Id, percentage of identity.

[c] *Ψ*, pseudogene.

[d] ND, not detected.

REFERENCES

1. Thevenard B, Rasoava N, Fourcassié P, Monnet V, Boyaval P, Rul F. 2011. Characterization of *Streptococcus thermophilus* two-component systems: In silico analysis, functional analysis and expression of response regulator genes in pure or mixed culture with its yogurt partner, Lactobacillus delbrueckii subsp. bulgaricus. *Int J Food Microbiol* 151:171–181.
2. Throup JP, Koretke KK, Bryant AP, Ingraham KA, Chalker AF, Yigong G, Marra A, Wallis NG, Brown JR, Holmes DJ, Rosenberg M, Burnham MKR. 2000. A genomic analysis of two-component signal transduction in *Streptococcus pneumoniae*. *Mol Microbiol* 35:566–576.
3. Buckley SJ, Timms P, Davies MR, McMillan DJ. 2018. In silico characterisation of the two-component system regulators of *Streptococcus pyogenes*. *PLoS One* 13:e0199163.
4. Lévesque CM, Mair RW, Perry JA, Lau PCY, Li YH, Cvitkovitch DG. 2007. Systemic inactivation and phenotypic characterization of two-component systems in expression of *Streptococcus mutans* virulence properties. *Lett Appl Microbiol* 45:398–404.
