# Supplementary material for: The CovRS Environmental Sensor Directly Controls the ComRS Signaling System To Orchestrate Competence Bimodality in Salivarius Streptococci
Source: mBio. 2022 Jan 4;13(1):e03125-21. doi: 10.1128/mbio.03125-21 (PMC8725580; doi:10.1128/mbio.03125-21)
Supplement: TABLE S2 [file mbio.03125-21-st002.pdf]

Table S2. Down-regulated genes in CovS$_{T287A}$ and CovR$_{D53E}$ mutants belonging to the ComR regulon

Locus tag	Strand	Gene	Description	Regulon ComR[a]	Regulon ComX[b]	Normalized ratio[c]	Normalized ratio[c]	ComR/X box[d]
				ΔcomR / WT	*ΔcomX* / WT	T287A / WT	D53E / WT	
HSISS4_00217	+	*comR*	Pheromone-responsive transcriptional regulator ComR			0.47	0.52	
**Core regulon of ComR**								
Locus comX								
HSISS4_00102	+	*comX*	Competence-specific sigma factor ComX	+		0.32	0.63	ComR
Locus 00176-00180								
HSISS4_00176	+		ABC transporter, ATP-binding protein	+		0.12	0.07	ComR
HSISS4_00177	+		ABC transporter permease protein	+		0.11	0.09	
HSISS4_00178	+	*pncB*	Nicotinate phosphoribosyltransferase	+		0.75	1.49	
HSISS4_00179	+	*nadE*	NAD synthetase	+		0.81	1.43	
HSISS4_00180	+		Transporter	+		0.46	1.57	
Locus comA								
HSISS4_00218	+	*comA*	Bacteriocin ABC transporter	+		0.04	0.03	ComR
Locus bacteriocin BlpK								
HSISS4_01581	-	*blpI*	Immunity protein	+		0.45	0.43	
HSISS4_01582	-		hypothetical protein	+		0.11	0.32	
HSISS4_01584	-		Biogenesis protein DsbD, protein-disulfide reductase	+		0.09	0.07	ComR
HSISS4_01585	-		hypothetical protein	+		0.19	0.04	
HSISS4_01586	-	*blpK*	Bacteriocin BlpU	+		0.07	0.07	ComR
Locus bacteriocin SlvV								
HSISS4_01594	-	*slvV*	putative bacteriocin SlvV	+		0.08	0.12	ComR

Locus bacteriocin SlvW								
HSISS4_01647	-	*comEB*	dCMP deaminase/Late competence protein ComEB	+		0.89	0.98	
HSISS4_01648	-	*pepP*	Aminopeptidase YpdF (MP-, MA-, MS-, AP-, NP- specific)	+		0.88	1.18	
HSISS4_01649	-		hypothetical protein	+		0.12	0.14	
HSISS4_01650	-		peptide ABC transporter ATP binding protein	+		0.19	0.24	
HSISS4_01651	-	*blpG*	hypothetical protein	+		0.14	0.12	
HSISS4_01652	-		hypothetical protein	+		0.08	0.41	
HSISS4_01653	-	*slvW*	putative bacteriocin SlvW	+		0.10	0.39	ComR
Locus bacteriocin SlvX								
HSISS4_01664	-		hypothetical protein	+		0.11	0.06	
HSISS4_01665	-	*slvX*	putative bacteriocin SlvX	+		0.04	0.05	ComR
Locus bacteriocin SlvYZ								
HSISS4_01742	+	*slvY*	putative bacteriocin SlvY	+		0.12	0.10	ComR
HSISS4_01743	+	*slvZ*	putative bacteriocin SlvZ	+		0.09	0.17	
HSISS4_01744	+		hypothetical protein	+		0.11	0.12	
**Accessory regulon of ComR**								
**Core regulon of ComX**								
HSISS4_00029	-	*cbpD1*	putative peptidoglycan hydrolase	+	+	0.44	0.34	ComX
HSISS4_00295	+	*comFA*	Late competence protein ComFA, DNA transporter ATPase	+	+	0.31	0.35	ComX
HSISS4_00296	+	*comFC*	Late competence protein ComFC, phosphoribosyltransferase domain	+	+	0.30	0.21	
HSISS4_00676	-	*pilD*	Late competence protein ComC, processing protease	+	+	0.36	0.78	ComX
HSISS4_01041	-	*dprA*	DNA recombination-mediator protein A	+	+	0.46	0.63	ComX
HSISS4_01377	-	*radC*	DNA repair protein RadC	+	+	0.43	0.36	ComX
HSISS4_01466	-	*comEC*	Late competence protein ComEC, DNA transport	+	+	0.30	0.20	

HSISS4_01467	-	*comEA*	Late competence protein ComEA, DNA receptor	+	+	0.35	0.20	ComX
HSISS4_01746	-	*ssbA*	ssDNA binding protein	+	+	0.29	0.22	ComX
HSISS4_01759	-	*ackA*	Acetate kinase	+	+	1.43	1.81	
HSISS4_01760	-		Adenine-specific methyltransferase	+	+	0.79	1.33	
HSISS4_01761	-	*comGG*	Late competence protein ComGG	+	+	0.33	0.49	
HSISS4_01762	-	*comGF*	Late competence protein ComGF, access of DNA to ComEA	+	+	0.24	0.18	
HSISS4_01763	-	*comGE*	Late competence protein ComGE	+	+	0.32	0.18	
HSISS4_01764	-	*comGD*	Late competence protein ComGD, access of DNA to ComEA	+	+	0.29	0.20	
HSISS4_01765	-	*comGC*	Late competence protein ComGC, access of DNA to ComEA	+	+	0.29	0.26	
HSISS4_01766	-	*comGB*	Late competence protein ComGB, access of DNA to ComEA	+	+	0.27	0.18	
HSISS4_01767	-	*comGA*	Late competence protein ComGA, access of DNA to ComEA	+	+	0.17	0.06	ComX
**Others - regulon ComX**								
HSISS4_00633	+		bacterial seryl-tRNA synthetase related	+	+	0.92	2.35	
HSISS4_00661	+		hypothetical protein	+	+	0.77	0.70	
HSISS4_00746	-		hypothetical protein	+	+	0.62	1.84	
HSISS4_00747	-		Polyphosphate(polyP) polymerase domain protein	+	+	0.55	2.03	
HSISS4_00855	+	*mur1B*	N-acetylmuramoyl-L-alanine amidase	+	+	0.69	0.37	
HSISS4_01277	+		Choline binding protein A	+	+	0.42	0.91	
HSISS4_01524	-		YoeB toxin protein	+	+	0.89	2.40	
HSISS4_01525	-		YefM protein (antitoxin to YoeB)	+	+	0.37	2.05	
**Others - regulon ComR**								
HSISS4_00104	+		hypothetical protein	+		0.16	0.05	

HSISS4_00301	-	*recX*	Regulatory protein recX	+		0.25	1.82
HSISS4_00697	+	*citB*	Aconitate hydratase	+		1.62	0.89
HSISS4_00698	+	*gltA*	Citrate synthase	+		1.74	0.88

[a] Down-regulated genes (red, ≥ 5-fold) in the Δ*comR* mutant (Δ*comR/*WT*)* that were previously reported (1). + denotes activation by ComR.

[b] Down-regulated genes (red, ≥ 5-fold) in the Δ*comX* mutant (ΔcomX/WT) that are belonging to the ComR regulon (1). + denotes activation by ComX.

[c] Ratio of normalized $CovS_{T287A}$ reads vs normalized reads of WT (T287A/WT) or $CovR_{D53E}$ vs WT (D53E/WT). Red, down-regulation > 5-fold, dark pink, between 2 and 5-fold, and light pink, between 1.5 and 2-fold.

[d] Mapping of upstream ComR (R)- and ComX (X)-boxes. Blue denotes genes belonging to the core regulon of ComR (8 loci) (1). Yellow denotes genes belonging to the core regulon of ComX that are indirectly controlled by ComR.

REFERENCE

1. Mignolet J, Fontaine L, Sass A, Nannan C, Mahillon J, Coenye T, Hols P. 2018. Circuitry Rewiring Directly Couples Competence to Predation in the Gut Dweller *Streptococcus salivarius*. *Cell Rep* 22:1627–1638.
