# Supplementary material for: The CovRS Environmental Sensor Directly Controls the ComRS Signaling System To Orchestrate Competence Bimodality in Salivarius Streptococci
Source: mBio. 2022 Jan 4;13(1):e03125-21. doi: 10.1128/mbio.03125-21 (PMC8725580; doi:10.1128/mbio.03125-21)
Supplement: FIG S4 [file mbio.03125-21-sf004.pdf]

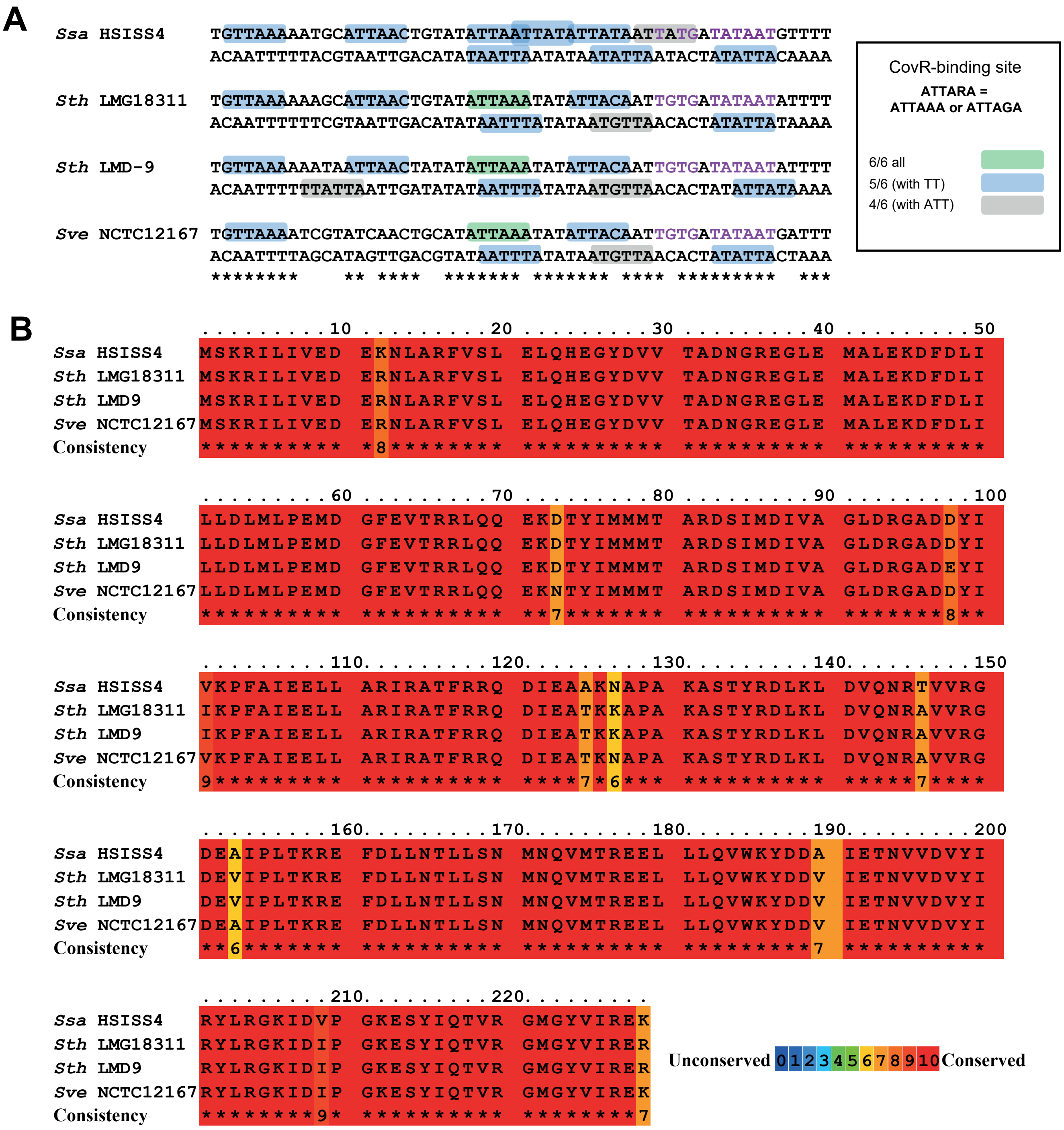
A
Ssa HSISS4
TGTTAAAAATGCATTAACTGTATATTAATTATATTATAATTATGATATAATGTTTT
ACAATTTTTACGTAATTGACATATAATTAATATAATATTAATACTATATTACAAAA
Sth LMG18311
TGTTAAAAAAGCATTAACTGTATATTAAATATATTACAATTGTGATATAATATTTT
ACAATTTTTTCGTAATTGACATATAATTTATATAATGTTAACACTATATTATAAAA
Sth LMD-9
TGTTAAAAAATAATTAACTATATATTAAATATATTACAATTGTGATATAATATTTT
ACAATTTTTTATTAATTGATATATAATTTATATAATGTTAACACTATATTATAAAA
Sve NCTC12167
TGTTAAAATCGTATCAACTGCATATTAAATATATTACAATTGTGATATAATGATTT
ACAATTTTAGCATAGTTGACGTATAATTTATATAATGTTAACACTATATTACTAAA
CovR-binding site
ATTARA =
ATTAAA or ATTAGA
6/6 all
5/6 (with TT)
4/6 (with ATT)
B
Ssa HSISS4
Sth LMG18311
Sth LMD9
Sve NCTC12167
Consistency
Unconserved
0 1 2 3 4 5 6 7 8 9 10
Conserved
