# Supplementary material for: The CovRS Environmental Sensor Directly Controls the ComRS Signaling System To Orchestrate Competence Bimodality in Salivarius Streptococci
Source: mBio. 2022 Jan 4;13(1):e03125-21. doi: 10.1128/mbio.03125-21 (PMC8725580; doi:10.1128/mbio.03125-21)
Supplement: TABLE S3 [file mbio.03125-21-st003.pdf]

## Table S3A. List of bacterial strains used in this study

| Strain | Characteristics | Reference/source |
|---|---|---|
| ***Escherichia coli*** | | |
| TOP10 | *mcrA*, Δ(*mrr-hsd*RMS-*mcrBC*), Phi80*lacZ(del)M15*, Δ*lacX74*, *deoR*, *recA1*, *araD139*, Δ(*ara-leu*)7697, *galU*, *galK*, *rpsL(SmR)*, *endA1*, *nupG* | Invitrogen, CA |
| ***Streptococcus thermophilus*** | | |
| LMG 18311 | Wild-type milky product isolate | BCCM |
| LMD-9 | Wild-type milky product isolate | ATCC |
| LF146 | LMG18311 *(blpU-blpX)*::P$_{comR}$-*luxAB* | (1) |
| LF150 | LMG18311 *(blpU-blpX)*::P$_{comR}$-*luxAB* Δ*covRS::lox72* | This work |
| CB009 | LMG18311 *(blpU-blpX)*::P$_{comX}$-*luxAB* | (1) |
| LF151 | LMG18311 *(blpU-blpX)*::P$_{comX}$-*luxAB* *covRS:kan* | This work |
| LF152 | LMG18311 *(blpU-blpX)*::P$_{comX}$-*luxAB* *covRS:lox72* | This work |
| LF153 | LMG18311 *(blpU-blpX)::P$_{comX}$-luxAB* pMG36ET | This work |
| LF154 | LMG18311 *(blpU-blpX)::P$_{comX}$-luxAB* Δ*covRS::lox72* pMG36ET | This work |
| LF155 | LMG18311 *(blpU-blpX)::P$_{comX}$-luxAB* Δ*covRS::lox72* pMG36ET-*covRS* | This work |
| CB001 | LMD-9 (*blpU-blpX*)::P$_{comX}$-*luxAB* | (2) |
| LF123 | LMD-9 (*blpU-blpX*)::P$_{comR}$-*luxAB* | (3) |
| LF156 | LMD-9 (*blpU-blpX*)::P$_{comX}$-*luxAB* *covRS:kan* | This work |
| LF157 | LMD-9 (*blpU-blpX*)::P$_{comX}$-*luxAB* *covRS:lox72* | This work |
| LF158 | LMD-9 (*blpU-blpX*)::P$_{comR}$-*luxAB* Δ*covRS::lox72* | This work |
| LF159 | LMG18311 *(blpU-blpX)::P$_{comX}$-luxAB* Δ*covRS::covRS*$_{\text{LMD-9}}$ | This work |
| LF160 | LMD-9 (*blpU-blpX*)::P$_{comX}$-*luxAB* Δ*covRS::covRS*$_{\text{LMG18311}}$ | This work |
| LF161 | LMG18311 *(blpU-blpX)::P$_{comX}$-luxAB* *covR*$_{\text{D98E}}$-*kan* | This work |
| LF162 | LMD-9 (*blpU-blpX*)::P$_{comX}$-*luxAB* *covR*$_{\text{E98D}}$-*kan* | This work |

| | | |
|---|---|---|
| AK0042 | LMD-9 *SUC*::P$_{comX}$-*opt-gfp-spc*<br>*tRNA*$_{ser}$::P$_{xyl2}$-*covR*$_{E98D}$-*cat* | This work |
| ***Streptococcus salivarius*** | | |
| HSISS4 | Wild-type gastro-intestinal tract isolate | (4) |
| AK0001 | HSISS4 *tRNA*$_{thr}$::*P*$_{comX}$-*luxAB-spc* | This work |
| AK0002 | HSISS4 *tRNA*$_{thr}$::*P*$_{comX}$-*luxAB-spc*<br>*tRNA*$_{ser}$::*P*$_{xyl2}$-*comS-cat* | This work |
| AK0003 | HSISS4 *tRNA*$_{thr}$::*P*$_{comX}$-*luxAB-spc*<br>*tRNA*$_{ser}$::*P*$_{xyl2}$-*xip-cat* | This work |
| AK0004 | HSISS4 *tRNA*$_{thr}$::*P*$_{comX}$-*luxAB-spc*<br>*tRNA*$_{ser}$::*P*$_{xyl2}$-*comR-cat* | This work |
| AK0005 | HSISS4 *tRNA*$_{thr}$::*P*$_{comX}$-*luxAB-spc*<br>*tRNA*$_{ser}$::*P*$_{xyl2}$-*comR-P*$_{comS}$-*comS-cat* | This work |
| AK0006 | HSISS4 *tRNA*$_{thr}$::*P*$_{comX}$-*luxAB-spc*<br>*tRNA*$_{ser}$::*P*$_{xyl2}$-*comR-P*$_{comS}$-*xip-cat* | This work |
| AK0007 | HSISS4 *tRNA*$_{thr}$::*P*$_{comX}$-*luxAB-spc*<br>*tRNA*$_{ser}$::*P*$_{32}$-*cat-amiA3* | This work |
| AK0008 | HSISS4 *tRNA*$_{thr}$::*P*$_{comX}$-*luxAB-spc*<br>*amiACDEF*::*P*$_{xyl2}$-*amiACDEF-cat* | This work |
| AK0009 | HSISS4 *tRNA*$_{thr}$::*P*$_{comX}$-*luxAB-spc*<br>*pptAB*::*P*$_{F6}$-*lacI-P*$_{lac}$-*pptAB-cat* | This work |
| AK0010 | HSISS4 *tRNA*$_{thr}$::*P*$_{comR}$-*luxAB-spc* | This work |
| AK0011 | HSISS4 *tRNA*$_{thr}$::*P*$_{comR}$-*luxAB-spc*<br>*covR* ::*covR*$_{D53A}$-*cat* | This work |
| AK0012 | HSISS4 *tRNA*$_{thr}$::*P*$_{comR}$-*luxAB-spc*<br>*covR* ::*covR*$_{D53E}$-*cat* | This work |
| AK0013 | HSISS4 *tRNA*$_{thr}$::*P*$_{comR}$-*luxAB-spc*<br>*covS* ::*covS*$_{T287A}$-*cat* | This work |
| AK0014 | HSISS4 *tRNA*$_{thr}$::*P*$_{comX}$-*opt-gfp-spc* | This work |
| AK0015 | HSISS4 *tRNA*$_{thr}$::*P*$_{comX}$-*opt-gfp-spc*<br>*tRNA*$_{ser}$::*P*$_{xyl2}$-*comR-cat* | This work |
| AK0016 | HSISS4 *tRNA*$_{ser}$::*P*$_{xyl2}$-*opt-gfp-spc* | This work |
| AK0017 | HSISS4 *tRNA*$_{thr}$::*P*$_{comX}$-*opt-gfp-lox72*<br>*tRNA*$_{ser}$::*P*$_{xyl2}$-*comR-lox72* | This work |
| AK0018 | HSISS4 *tRNA*$_{thr}$::*P*$_{comX}$-*opt-gfp-lox72*<br>*tRNA*$_{ser}$::*P*$_{xyl2}$-*comR-lox72*<br>*ΔcomS*::*cat* | This work |
| AK0019 | HSISS4 *tRNA*$_{thr}$::*P*$_{comX}$-*opt-gfp-lox72*<br>*tRNA*$_{ser}$::*P*$_{xyl2}$-*comR-lox72*<br>*Δopp*::*cat* | This work |
| AK0020 | HSISS4 *tRNA*$_{thr}$::*P*$_{comX}$-*opt-gfp-lox72*<br>*tRNA*$_{ser}$::*P*$_{xyl2}$-*comR-lox72*<br>*ΔpptAB*::*erm* | This work |

| | | |
|---|---|---|
| AK0021 | HSISS4 *tRNA*$_{thr}$*::P*$_{comX}$*-opt-gfp-lox72*<br>*tRNA*$_{ser}$*::P*$_{xyl2}$*-comR-lox72*<br>*pptAB::P*$_{F6}$*-lacI-P*$_{lac}$*-pptAB-cat* | This work |
| AK0022 | HSISS4 *tRNA*$_{thr}$*::P*$_{comR}$*-opt-gfp-spc* | This work |
| AK0023 | HSISS4 *tRNA*$_{thr}$*::P*$_{comX}$*-opt-gfp-lox72*<br>*tRNA*$_{ser}$*::P*$_{xyl2}$*-comR-lox72*<br>*ΔcomS::cat*<br>*tnpII:: P*$_{xyl2}$*-comS-spc* | This work |
| AK0024 | HSISS4 *tRNA*$_{thr}$*::P*$_{comX}$*-opt-gfp-lox72*<br>*tRNA*$_{ser}$*::P*$_{xyl2}$*-comR-lox72*<br>*ΔcomS::cat*<br>*tnpII:: P*$_{xyl2}$*-xip-spc* | This work |
| AK0025 | HSISS4 *tRNA*$_{thr}$*::P*$_{comX}$*-opt-gfp-lox72*<br>*tRNA*$_{ser}$*::P*$_{xyl2}$*-comR-lox72*<br>*ΔcomS::cat*<br>*tnpII::* P$_{32}$*-comS-spc* | This work |
| AK0026 | HSISS4 P$_{comR}$*-comR-opt-gfp*$^{+}$*-spc* | This work |
| AK0027 | HSISS4 *tRNA*$_{thr}$*::*P$_{F6}$*-lacI-spc* | This work |
| AK0028 | HSISS4 *tRNA*$_{thr}$*::*P$_{F6}$*-lacI-spc*<br>*tRNA*$_{ser}$*::*P$_{lac}$*-dcas9-cat* | This work |
| AK0029 | HSISS4 *tRNA*$_{thr}$*::*P$_{F6}$*-lacI-lox72*<br>*tRNA*$_{ser}$*::*P$_{lac}$*-dcas9-lox72* | This work |
| AK0030 | HSISS4 *tRNA*$_{thr}$*::*P$_{F6}$*-lacI-lox72*<br>*tRNA*$_{ser}$*::*P$_{lac}$*-dcas9-lox72*<br>*tnpII::P*$_{comX}$*-luxAB-spc* | This work |
| AK0031 | HSISS4 *tRNA*$_{thr}$*::*P$_{F6}$*-lacI-lox72*<br>*tRNA*$_{ser}$*::*P$_{lac}$*-dcas9-lox72*<br>*tnpII::P*$_{comR}$*-luxAB-spc* | This work |

| | | |
|---|---|---|
| AK0032 | HSISS4 *tRNA*$_{thr}$::P$_{F6}$-*lacI-lox72*<br>*tRNA*$_{ser}$::P$_{lac}$-*dcas9-lox72*<br>*tnpII::P*$_{comR}$-*luxAB-spc* | This work |
| AK0033 | HSISS4 *tRNA*$_{thr}$::P$_{F6}$-*lacI-lox72*<br>*tRNA*$_{ser}$::P$_{lac}$-*dcas9-lox72*<br>*tnpII::P*$_{comX}$-*luxAB-lox72* | This work |
| AK0034 | HSISS4 *tRNA*$_{thr}$::P$_{F6}$-*lacI-lox72*<br>*tRNA*$_{ser}$::P$_{lac}$-*dcas9-lox72*<br>*tnpII::P*$_{comR}$-*luxAB-lox72* | This work |
| AK0035 | HSISS4 *tRNA*$_{thr}$::P$_{F6}$-*lacI-lox72*<br>*tRNA*$_{ser}$::P$_{lac}$-*dcas9-lox72*<br>*tnpII::P*$_{comX}$-*opt-gfp-lox72* | This work |
| AK0036 | HSISS4 *GOR::P*$_{3}$-*g*_1(*luc*) | This work |
| AK0037 | HSISS4 *tRNA*$_{thr}$::P$_{F6}$-*lacI-lox72*<br>*tRNA*$_{ser}$::P$_{lac}$-*dcas9-lox72*<br>*tnpII::P*$_{32}$-*luxAB-spc*<br>*GOR::P3-g_3(P*$_{32}$*)-erm* | This work |
| AK0038 | HSISS4 *tRNA*$_{thr}$::P$_{F6}$-*lacI-lox72*<br>*tRNA*$_{ser}$::P$_{lac}$-*dcas9-lox72*<br>*tnpII::P*$_{comX}$-*luxAB-lox72*<br>*GOR*::P$_{3}$-*g_19(*P$_{covRS*}$)-*erm*<br>P$_{covRS}$::P$_{covRS*}$-*cat* | This work |
| AK0039 | HSISS4 *tRNA*$_{thr}$::P$_{F6}$-*lacI-lox72*<br>*tRNA*$_{ser}$::P$_{lac}$-*dcas9-lox72*<br>*tnpII::P*$_{comR}$-*luxAB-lox72*<br>*GOR*::P$_{3}$-*g_19(*P$_{covRS*}$)-*erm*<br>P$_{covRS}$::P$_{covRS*}$-*cat* | This work |
| AK0040 | HSISS4 *tRNA*$_{thr}$::P$_{F6}$-*lacI-lox72*<br>*tRNA*$_{ser}$::P$_{lac}$-*dcas9-lox72*<br>*tnpII::P*$_{comX}$-*opt-gfp-lox72*<br>*GOR*::P$_{3}$-*g_19(*P$_{covRS*}$)-*erm*<br>P$_{covRS}$::P$_{covRS*}$-*cat* | This work |
| FV0001 | HSISS4 *P*$_{comR}$-*comR:luxAB-lox72* | This work |
| FV0002 | HSISS4 *P*$_{comR}$-*comR:luxAB-lox72*<br>Δ*covs::cat* | This work |
| FV0003 | HSISS4 *P*$_{comR}$-*comR:luxAB-lox72*<br>Δ*ciaRH::cat* | This work |

| | | |
|---|---|---|
| FV0004 | HSISS4 $P_{comR}$-*comR:luxAB-lox72* Δ*spaRK::cat* | This work |
| FV0005 | HSISS4 $P_{comR}$-*comR:luxAB-lox72* Δ*vicK::cat* | This work |
| FV0006 | HSISS4 $P_{comR}$-*comR:luxAB-lox72* Δ*TCS06::cat* | This work |
| FV0007 | HSISS4 $P_{comR}$-*comR:luxAB-lox72* Δ*BceRS::cat* | This work |
| FV0008 | HSISS4 $P_{comR}$-*comR:luxAB-lox72* Δ*LiaSR::cat* | This work |
| FV0009 | HSISS4 $P_{comR}$-*comR:luxAB-lox72* Δ*TCS09::cat* | This work |
| FV0010 | HSISS4 $P_{comR}$-*comR:luxAB-lox72* Δ*TCS10::cat* | This work |
| FV0011 | HSISS4 $P_{comR}$-*comR:luxAB-lox72* Δ*vncRS::cat* | This work |
| FV0012 | HSISS4 $P_{comR}$-*comR:luxAB-lox72* Δ*fasB::cat* | This work |
| FV0013 | HSISS4 $P_{comR}$-*comR:luxAB-lox72* Δ*TCS13::cat* | This work |
| FV0014 | HSISS4 $P_{comR}$-*comR:luxAB-lox72* Δ*TCS14::cat* | This work |
| JM0001 | HSISS4 Δ*comR* | (5) |
| JM1125 | HSISS4 $tRNA_{ser}$::$P_{xyl2}$-$gfp^{+}$-*spc* | (6) |
| JM1016 | HSISS4 $tRNA_{ser}$::$P_{xyl2}$-*comR-spc* | (5) |
| JM1019 | HSISS4 $tRNA_{thr}$::$P_{comS}$-luxAB-cat | (5) |
| JM1020 | HSISS4 $tRNA_{thr}$::$P_{comX}$-luxAB-cat | (5) |

[a] *cat, spc, erm, kan,* indicate resistance to chloramphenicol, spectinomycin, erythromycin, kanamycin resistance and *lox72* stands for the lox scar.
[b] BCCM, Belgian Coordinated Collections of Microorganisms, LMG Laboratory of Microbiology and Genetics, Ghent, Belgium.
[c] ATCC, American Type Culture Collection, Rockville, MD.

**Table S3B. List of plasmids used in this study**

| Plasmid | Characteristics | Reference/source |
|---|---|---|
| pBAD-comR-ST | pBAD*hisA* derivative encoding $ComR_{HSISS4}$ fused to a C-terminal StreptagII | (5) |
| pBAD-$covR_{WT}$-$ST_{N\text{-}ter}$ | pBAD-comR-ST derivative encoding HSISS4 $CovR_{WT}$ fused to a N-terminal StreptagII | This work |
| pBAD-$covR_{D53A}ST_{N\text{-}ter}$ | pBAD-comR-ST derivative encoding $CovR_{D53A}$ fused to a N-terminal StreptagII | This work |
| pBAD-$covR_{D53E}ST_{N\text{-}ter}$ | pBAD-comR-ST derivative encoding $CovR_{D53E}$ fused to a N-terminal StreptagII | This work |
| pGhostcre | Thermosensitive replication origin vector in *S. salivarius*, encoding the Cre recombinase; *erm* | (7) |
| pGIUD0855erm | pUC18 derivative containing the *erm* gene | (7) |
| pJUDspecmut1-gfp+ter | Terminator associated-*gfp* ORF | (5) |
| pJIMcat | pJIM4900 derivative with a *cat* cassette | (5) |
| pMG36ET | Expression vector; *erm* | (8) |
| pMG36ET-covRS | pMG36ET derivative encoding the *covRS* operon under the $P_{32}$ constitutive promoter; *erm* | This work |
| pJWV102-PL-dcas9 | Plasmid encoding a dead Cas9 under the control of a lactose inducible promoter | (9) |
| pPEPY-PF6-lacI | Plasmid harboring a *lacI* repressor under the control of the $P_{F6}$ promoter, optimized for *S. pneumoniae* | (9) |
| pPEPX-P3-sgRNAluc | Plasmid harboring a $P_3$ constitutive promoter fused to a guide targeting the *luc* firefly luciferase gene together with a dcas9-handle and a terminator, *spc* | (9) |

**Table S3C. List of oligonucleotides used in this study**

| Name | Sequence (5'-3') |
|---|---|
| AK44 | ATGAAAAAAAGCAAATGG |
| AK50 | TGGGATTTATCTTCCTTAACTTATAGGGGTAACAC |
| AK52 | TAACTAACAGTTAAAAGGAGTTTTATTTTGATCACTATCTTGCCTTATTTTG |
| AK55 | AAATTGAGAGGTATAAATCAAGATTCCAAAGTAAACAGAAA |
| AK56 | GTATGTAAGCAAAAAGTTTCCAAATTTCATGAGACACTCCTTTATTTC |
| AK57 | CTGAAGAGTGTAATCATCAAG |
| AK58 | ATGAAAAAAAGTAAATGGTTGG |
| AK59 | GCCAACCATTTACTTTTTTTCATATTTACCTCCTTTGATTTAAGTG |
| AK60 | AAAGATCTTGAGAAACACGTTAAATAATTCACGTTACTAAAGGGA |
| AK61 | TTATTTAACGTGTTTCTCAAGAT |
| AK62 | ATTATCATCTGATGACATACAAAATG |
| AK69 | CTGTTAAAACCATCTTCTTTTTAATTACTTAAGGAAGATAAATCCCATAAG |
| AK70 | GCTAACCATTTGCTTTTTTTCATTTCACGTTACTAAAGGGAA |
| AK71 | GTAATTAAAAAGAAGATGGTTTTAACAGCTTTTATTTAACGTGTTTCTCAAGATC |
| AK87 | CTTTTGAAAAATTGAGAGGTATAAATCAAAGTATAGATTTCATTGCTGG |
| AK88 | CTTATGGGATTTATCTTCCTTATAAATAATAAAAAAGCCGGATTAATAAT |
| AK91 | CCTTATGGGATTTATCTTCCTTACTATTAGGTATATTCCATGTG |
| AK105 | CTTTTGAAAAATTGAGAGGTATAAATCAAATTAATAGTTTTAGCTATTAATCTTTTT |
| AK106 | CTTTTGAAAAATTGAGAGGTATAAATCAACTGCAGAAAATTACAATAAGG |
| AK107 | GTGGTTATTATTCAAATTGCAG |
| AK108 | CTTGATCTGCAATTTGAATAATAACCACTCCTTTGTTTATCCTCCTC |
| AK112 | CTTTTGAAAAATTGAGAGGTATAAATCAAGAATGGCGATTCCAAAG |
| AK121 | TGAACAATTGTTGCAATTTC |
| AK122 | CACCTATTAAATAAGTTAAAATAGAAAC |
| AK123 | CCGTCACCTTTTCTAATTTAAG |
| AK124 | GATATTTTCTCGTGATTGG |
| AK133 | CTATACTGAACCAAGACAAAG |
| AK134 | TACATTCCCTTTAGTAACGTGAATATCTTTTCTCTCTTTGGTATG |
| AK147 | GAAGGGACACTTTGTCTATTTCTGACTTATAGGGGTAACACTTAAAAAAG |
| AK148 | CTCCTCTCCTTTTCAGAAATAGACAAATTTTCACGTTACTAAAGGGAATGTA |
| AK151 | ATGAAAAAACTAAAATTATTTACAC |
| AK152 | AAGACAACCTGCAAAATAAG |
| AK153 | CTTGCCTTATTTTGCAGGTTGTCTTTAAGGAAGATAAATCCCATAAGG |
| AK169 | CGTTTCTATTTTAACTTATTTAATAGGTGCTACACTACCTAATAATTTATC |
| AK170 | GCTTAAATTAGAAAAGGTGACGGAGTGCAAAAAAAGATATAATGG |
| AK184 | GTTTCAGAAACCTTTATACAAG |
| AK187 | CCTTATGGGATTTATCTTCCTTATTAAAGCTCAAGTTGAGGCG |
| AK188 | TACATTCCCTTTAGTAACGTGAAATCAATTTATAAGAGACTTAGAGTG |
| AK189 | CGTACAGTTTTGGTGATTTC |
| AK194 | CAACATAAGTTCCAAGAGAATAAGATCGAAATC |
| AK195 | CTCTTGGAACTTATGTTGCCTGAGATGGAC |
| AK222 | GTGAGAATAGTGTAAATAATTTTAGTTTTTTCAATTGATATGCCTCCTAAATTTTTATC |
| AK245 | CATAAGGGCCAAGAGAATAAGATCGAAATC |
| AK246 | CTCTTGGCCCTTATGTTGCCTGAGATGG |

| | |
|---|---|
| AK251 | CGGCGCACGCAATTCATGACTGACGTC |
| AK252 | GAATTGCGTGCGCCGATTGCGGTTATCAAG |
| AK262 | CATTTTGACTGCTATCTTTG |
| AK263 | CAAATACTAATCAAATGATATTTATC |
| AK264 | TTAAGTAGGAGTGCTTAATTC |
| AK265 | AAATGAGTTATCCTTTCTCC |
| AK266 | TTAAAATATAGATAAATATCATTTGATTAGTATTTGTTCACGTTACTAAAGGGAATGTA |
| AK267 | GTTTGAATTAAGCACTCCTACTTAATAAGGAAGATAAATCCCATAAGG |
| AK269 | CAGTATTTCCTTACACTAGG |
| AK270 | CCTATGACCTATACGAAATC |
| AK273 | GTTTGAATTAAGCACTCCTACTTAATCTATAAAATGCATACTGTTATTG |
| AK287 | GAACTGCGGGTGACTCCACATGGTTAATTCCTCCTGTTAG |
| AK288 | CTAACAGGAGGAATTAACCATGTGGAGTCACCCGCAGTTCGAGAAAGGTGCTGCTGGTAGCAAACGCATTTTGATTGTTG |
| AK290 | GCCAAGCTTCTAGAATTCTCATTTTTCACGGATAACGTAACC |
| AK291 | GGTTACGTTATCCGTGAAAAATGAGAATTCTAGAAGCTTGGC |
| AK298 | ACAATTTTAAAAATCTATATTATTATATC |
| AK299 | CATAATAAGTATTTACCACTTTC |
| AK300 | AAAAATAATGAGCACTTAAAAGAATG |
| AK301 | CATGAAGGCCTCCTTTCTATC |
| AK302 | CCTGTAGTTCCCTTACATAC |
| AK303 | TTCCATTTCTTGAGGCGAG |
| AK304 | TTGATACAAAGGAAGGTCTTTC |
| AK305 | CAAGAGACACTCCTTTATTTC |
| AK326 | CAAGAGACACTCCTTTATTTC |
| AK336 | CTTTTGAAAAATTGAGAGGTATAAATCAATAGATAGTAGCGGTGAATCTAG |
| AK337 | CCTTATGGGATTTATCTTCCTTATTATTATTATTGTCCACTTTCC |
| AK337 | CCTTATGGGATTTATCTTCCTTATTATTATTATTGTCCACTTTCC |
| AK340 | CTGTTAAAACCATCTTCTTTTTAATTACTGAACGCTCTCTACTAGAGTC |
| AK341 | CCTTATGGGATTTATCTTCCTTATTAGTCACCTCCTAGCTGAC |
| AK347 | TCTCCTTTACCAGCAGCACCTAAACCATCTGCCGCTTTAC |
| AK348 | GGTGCTGCTGGTAAAGGAGAAGAATTGTTTACTG |
| AK349 | AAAAATAATGAGCACTTAAAAGAATG |
| AK350 | CCTGTAGTTCCCTTACATAC |
| AK351 | TTGATACAAAGGAAGGTCTTTC |
| AK354 | TGCATGATCTACGTGCGTCAC |
| AK355 | CAACAGCGGTCTACTGAATCTG |
| AK361 | TTGATATGCCTCCTAAATTTTTATC |
| AK362 | CGTTTGAATTAAGCACTCCTACTTAAGTCCTCGGGATATGATAAG |
| AK362 | CGTTTGAATTAAGCACTCCTACTTAAGTCCTCGGGATATGATAAG |
| AK364 | GGTAGATAAAAATTTAGGAGGCATATCAAATGAAATTTGGAAACTTTTTGC |
| AK365 | CGTTTGAATTAAGCACTCCTACTTAAAGTATAGATTTCATTGCTGG |
| AK448 | CGTTTGAATTAAGCACTCCTACTTAAACTTATAGGGGTAACACTTAAAAAAG |
| AK452 | TGAGTGAATGGTTTCAATTG |
| AK453 | AACGGGTTCAGAAGAATTTG |
| AK455 | CTGGCTTCAATTGAAACCATTCACTCATTCACGTTACTAAAGGGAATGTA |
| AK458 | GTAGCAACACTCTTGTTTAAAG |
| AK459 | GTGGCTGAATTATCAAAATAAATC |

| | |
|---|---|
| AK462 | GAACAAAAACTCATGGATCCCTCCTAAGGAAGATAAATCCCATAAGG |
| AK463 | GAGGGATCCATGAGTTTTTG |
| AK464 | CGTCACAAATTCTTCTGAACCCGTTGGTCGACAGATCTCCATTC |
| AK472 | AAGTTTAAATAAGGCTAGTCCGTTATCAACTTG |
| AK473 | TATAGTTATTATACCAGGGGGAC |
| AK474 | CGGACTAGCCTTATTTAAACTTGCTATGCTGTTTCCAGCATAGCTCTTAAACAAATTTTTATCTACCTAGTATATAGTTATTATACCAGGGGGAC |
| AK475 | CGGACTAGCCTTATTTAAAC |
| AK476 | GTCCCCCTGGTATAATAACTATA |
| AK505 | GTTTGAATTAAGCACTCCTACTTAAGATTCCAAAGTAAACAGAAATAAAAAAAC |
| AK509 | CAAAAGATCTAAAGAGGAGAAAGGATCTATGCTTAAATTAGAAAAGGTGAC |
| AK510 | AGATCCTTTCTCCTCTTTAG |
| AK511 | GAACGCTCTCTACTAGAGTC |
| AK512 | GTGACTCTAGTAGAGAGCGTTCTAGATAGTAGCGGTGAATC |
| AK534 | CGGACTAGCCTTATTTAAACTTGCTATGCTGTTTCCAGCATAGCTCTTAAACAACTAAAAATGTTTGACAATTATAGTTATTATACCAGGGGGAC |
| AK537 | TTAATATGTCCTTGGAGAAC |
| AK538 | TTTTAGTTTGGTTTTTTGAATTAATTTTCATCAAAAAGAC |
| AK539 | ATTCAAAAAACCAAACTAAAAATGTTTGACAATTTTAAAAATC |
| AK544 | GAAACTGCTTAAATCAAACG |
| AK565 | GGCGAATCTAAAAATAAAGG |
| AK566 | TGGTAGCAGCTTAAATATGC |
| AK567 | CCAACTTGGCTTCATATCAC |
| AK568 | CTTGAAGCTAACCTTATTTC |
| AK569 | CTAAATAAAAGCATATTTAAGCTGCTACCAAGTATAGATTTCATTGCTGG |
| AK570 | GAAGAGTGATATGAAGCCAAGTTGGTTCACGTTACTAAAGGGAATG |
| AK575 | ATGAGCAAACGCATTTTAATTGTTGAAGATGAGAGAAACC |
| AK576 | CAACAATTAAAATGCGTTTGCTCATATTTACCTCCTTTGATTTAAGTG |
| AK612 | CCTTATGGGATTTATCTTCCTTATCATCTTTCACGGATAACG |
| AR901 | AACTATCATCCGTCATCCCAG |
| DD_DNDELCOVRS-1 | TACATTCCCTTTAGTAACGTGAAACACCTCAAATTGAAGGT |
| DD_DNDELCOVRS-2 | GGTTTTAAAGCATCCACTGCTGGCTCACC |
| DD_UPDELCOVRS-1 | TTGGAAATCCAGCTCCAGCACCGATATCC |
| DD_UPDELCOVRS-2 | CCTTATGGGATTTATCTTCCTTACAAAATGCGTTTGCTCAT |
| DF_COMS | TACATTCCCTTTAGTAACGTGAATAATAAGGAGTCACCATGTC |
| DF_SER | AAGCTGTTAAAACCATCTTC |
| DN.RV.LOX71 | TTCACGTTACTAAAGGGAATGTA |
| DN_F_BCERS_LOX71 | TACATTCCCTTTAGTAACGTGAAGTTGGCGATTGATTAATAAC |
| DN_F_CIARH_LOX71 | TACATTCCCTTTAGTAACGTGAATAGATAAGGCTAGAACCAGAC |
| DN_F_COVS_LOX71 | TACATTCCCTTTAGTAACGTGAAGCCTCAACTTGAGCTTTAAATC |
| DN_F_FASB_LOX71 | TACATTCCCTTTAGTAACGTGAAGGCCCTAATATGAATATATAC |
| DN_F_LIASR_LOX71 | TACATTCCCTTTAGTAACGTGAACGCAGGATGATGAATAAAGC |

| | |
|---|---|
| DN_F_SPARK_LOX71 | TACATTCCCTTTAGTAACGTGAACTTTAAATACAGATGACTCC |
| DN_F_TCS06_LOX71 | TACATTCCCTTTAGTAACGTGAATGAGGAGAAGTAGATAAATTTTC |
| DN_F_TCS09_LOX71 | TACATTCCCTTTAGTAACGTGAAGATGTGGTTTGATTATGACG |
| DN_F_TCS10_LOX71 | TACATTCCCTTTAGTAACGTGAATTTAAATATTTGACTAGGTG |
| DN_F_TCS13_LOX71 | TACATTCCCTTTAGTAACGTGAACAGTGAGATTATAAAAACATG |
| DN_F_TCS14_LOX71 | TACATTCCCTTTAGTAACGTGAAACACCAAAAGAAGATTAATG |
| DN_F_VICK_LOX71 | TACATTCCCTTTAGTAACGTGAAGAGATGTAATAGAAATGACC |
| DN_F_VNCRS_LOX71 | TACATTCCCTTTAGTAACGTGAAGGTCTTGCCCAAAAAGTAAAAAG |
| DN_R SS1-4 | TCATCACAATGGTCACATCT |
| DN_R_BCERS | CTACTTGTGTCATCATTTGC |
| DN_R_CIARH | GCCTAAAATAACGTATAATGAC |
| DN_R_COVS | TGAAAGAACGGTAGACACTG |
| DN_R_DIAG_DEL_COVS | CAAATTCTTCAATAGGTCTC |
| DN_R_DIAG_DEL_FASB | CCAAATTAACTAAAAAGGAGCG |
| DN_R_DIAG_DEL_VICK | ATCCGCTCCATCTTCATTAC |
| DN_R_DIAGDEL_BCERS | GTATTTCTGGTTTAGTGATG |
| DN_R_DIAGDEL_CIARH | CTGGCGTGATATCAATTTGG |
| DN_R_DIAGDEL_LIASR | ACAGCCATCACAAAACAAAG |
| DN_R_DIAGDEL_SPARK | CTTAAGGTAGACTTTGTTCA |
| DN_R_DIAGDEL_TCS06 | GTTTCACTTCTATCATTGCC |
| DN_R_DIAGDEL_TCS09 | ATATCAGAAGCAATTGTTCC |
| DN_R_DIAGDEL_TCS10 | GCAAAGATAACAATACGACC |
| DN_R_DIAGDEL_TCS13 | CAGAGATTGTATCTGGATTC |
| DN_R_DIAGDEL_TCS14 | TCTTAGCTGCTTTTTCTTCC |
| DN_R_DIAGDEL_VNCRS | GATATTTAACCTGTCTACTTG |
| DN_R_FASB | TTTTAGTAATCAGCATCACC |
| DN_R_LIASR | TAGCTGGTACTTATAGTCCG |
| DN_R_SPARK | GAAGCAATAACTGTAATGGC |
| DN_R_TCS06 | TCTCCTAAAATCTCCCTTC |
| DN_R_TCS09 | CCAGCGATTGAACTATAATC |
| DN_R_TCS13 | GGTTCATACTCTGTTTTATC |
| DN_R_TCS14 | GAATGATTTCCTGCATCTGG |
| DN_R_VICK | ATTGGACTCAATCAGATAGC |
| DN_R_VNCRS | AATGGTTTCCCTTCAGC |
| DN_R_VNCRS_2 | ACCACCTTTGCTAGCTG |

| | |
|---|---|
| DN-COVRS-2 | GGTTTTAAAGCATCCACTGCTGGCTCACC |
| DN-INSERTCOVRSKAN-F | GGGAAATATTCATTCTAATTGGTCAATCATGAGCACGTC |
| DR_SER | TTGGATAAGGTCTTGACTTC |
| DR_THR | TTGATTTATACCTCTCAATTT |
| F_LUX_ATG | ATGAAATTTGGAAACTTTTTGC |
| JB546 | GGTGTTGTATTCATCTTCAC |
| KAN OVL1 | TTGATTTTCGTTCGTGAATAC |
| KAN OVL2 | CCAATTAGAATGAATATTTCCC |
| LF-DN_LMGCOVRD98E_3'A | GTTTGGATCGTGGAGCTGATGAGTATATTATAAAACCATTTGCG |
| LF-PMGCOVRD9XBAI-F | AAGCTCTAGATTTATAAAAGAGGGTAAATACTTATTATGAGC |
| LF-PMGCOVSLMGPSTI-R | AAACTGCAGATAAATCTCTTGTAAATTGACTTAACC |
| LF-UP D9COVR_E98D-5'B | GTCATCAGCTCCACGATCCAAACCAGCAACAATATC |
| LF-UP LMGCOVR_D98E-5'B | CTCATCAGCTCCACGATCCAAACCAGCAACAATATC |
| LL61 | CATGGGAGTTCTCATCCTATAAATCAACCTCTTTGAACATA |
| LL150 | TACATTCCCTTTAGTAACGTGAA |
| MV13 | CACTTAAATCAAAGGAGGTAAATATGCCGACATTAGAAATAGCACAAAAAAAAC |
| MV23 | CACTTAAATCAAAGGAGGTAAATATGAAAAAACTAAAATTATTTACACTATTC |
| MV24 | CACTTAAATCAAAGGAGGTAAATATGATCACTATCTTGCCTTATTTTG |
| MV25 | CTTATGGGATTTATCTTCCTTAAATATATGGATATTTTGACATGG |
| MV29 | CACTTAAATCAAAGGAGGTAAATATGAACATAAAAGACAGCATTGGAC |
| MV30 | CTTATGGGATTTATCTTCCTTACCATTAAAAAAGTAGTGACATTTATG |
| ML38 | CTTTATCTACGTCAGTATAAATC |
| ML40 | TACATTCCCTTTAGTAACGTGAAAAATGGTGGTGACATAAATG |
| ML41 | CTTACGACGAAACAAAATTG |
| ML45 | TAAGGAAGATAAATCCCATAAGGTAAACATAAACATGCAAGGAG |
| ML46 | TTCACGTTACTAAAGGGAATGTAGAAAAGGCAATTGCGTTACC |
| OPP_S4_1 | TGTAGCACGTGCACAATCTT |
| OPP_S4_2CAT | CCTTATGGGATTTATCTTCCTTATCAAGGCCGTCACGACAAATA |
| OPP_S4_3CAT | TACATTCCCTTTAGTAACGTGAAAAAGAACAAAGTAAATAAGTCT |
| OPP_S4_6B | TCACACTGATGGTTTTTGCT |
| PB11 | CAGAAATAGACAAAGTGTCCCTTCCAGCATAG |
| PB12 | AATTTGTCTATTTCTGAAAAGGAGAGGAGGGG |
| R_SPEC | ATAGGATGAGAACTCCCATG |
| RPXZ9_ATG | CATATTTACCTCCTTTGATTTA |
| SEQCOVRS-F5 | TCCTTGAAGAAAGTCTTGAAGCTGC |
| UF_R-LUX | TAATTGAGGAGGTCTATGAG |

| | |
|---|---|
| UF_R-LUX-ATG | ATGAAATTTGGAAACTTTTTGC |
| UF_SER | CAAGATTAACCATGACCTTC |
| UF_THR | TGTCAAAGGATTAGGAAAAC |
| UP.FW.LOX66 | TAAGGAAGATAAATCCCATAAGG |
| UP.INSERTCOVRSKAN-R | GTATTCACGAACGAAAATCAAGAGTTAGTATAAATCTCTTG |
| UP_F_BCERS | ACTCTGACAAAGATCAAACC |
| UP_F_CIARH | GTTACTGAAATCTATGGTGG |
| UP_F_COVS | CTCTTGGACCTTATGTTGCC |
| UP_F_DIAG_DEL_COVS | CTCTCTTGAACTTCAACACG |
| UP_F_DIAG_DEL_FASB | GTCTATAATAAACATATTGAGG |
| UP_F_DIAG_DEL_VICK | TGTTACGGCTTTTGATGGTC |
| UP_F_DIAGDEL_BCERS | TGCGGTCTTCCAGTAATTTC |
| UP_F_DIAGDEL_CIARH | GATGGTGGTATTGAACTTGC |
| UP_F_DIAGDEL_LIASR | TTTTGACTCTGGGCCTGATC |
| UP_F_DIAGDEL_SPARK | CAATTCCAATGTATGTGAGC |
| UP_F_DIAGDEL_TCS06 | TTCAGGTATGGAAATGAGTC |
| UP_F_DIAGDEL_TCS09 | TAGATGGCAGCCCAATCTTG |
| UP_F_DIAGDEL_TCS10 | TCTTAGCTTTAGAGTCCTTC |
| UP_F_DIAGDEL_TCS13 | CATGCAACCTTGGCGTTTTG |
| UP_F_DIAGDEL_TCS14 | TGGTTTTCTACAGCCTTATG |
| UP_F_DIAGDEL_VNCRS | CTCTACGATCTTGTGAAGAG |
| UP_F_FASB | AGAGGTTTTATCCGTTTATC |
| UP_F_LIASR | TTGACCATCTGTTTGATATC |
| UP_F_SPARK | AATATCGAGTTACCCTAAGC |
| UP_F_TCS06 | ATGCTTAGAGAGGACCAAAC |
| UP_F_TCS09 | GTTCTTTCTCGTATGGGGTG |
| UP_F_TCS10 | TACAAGAAATAACAGACCCG |
| UP_F_TCS13 | GATTTGAAGAGCCAAGAAAG |
| UP_F_TCS14 | AAAAGTTGGAGTGTTTGTGG |
| UP_F_VICK | CAAGTCACACACCAATTATC |
| UP_F_VNCRS | GTTTATTGCTAACCCTTCTG |
| UP_R_BCERS_LOX66 | CCTTATGGGATTTATCTTCCTTATCCATGAAATCTTCAACAAG |
| UP_R_CIARH_LOX66 | CCTTATGGGATTTATCTTCCTTACTAGTAGTATTTTAATCATAGTTATC |
| UP_R_COVS_LOX66 | CCTTATGGGATTTATCTTCCTTAGTAATTTACTCATTTTTCACGG |
| UP_R_FASB_LOX66 | CCTTATGGGATTTATCTTCCTTATTTGTCCACCAATACGTAAG |
| UP_R_LIASR_LOX66 | CCTTATGGGATTTATCTTCCTTACTGTTTCTTCATTTGCGACG |

| | |
|---|---|
| UP_R_SPARK_LOX66 | CCTTATGGGATTTATCTTCCTTAGAGCCATAACCTTTCCTTTC |
| UP_R_TCS06_LOX66 | CCTTATGGGATTTATCTTCCTTACTAAACATGGTCACTTTCTC |
| UP_R_TCS09_LOX66 | CCTTATGGGATTTATCTTCCTTAGCTCGGTAATAGACTCTAAC |
| UP_R_TCS10_LOX66 | CCTTATGGGATTTATCTTCCTTAAATTTTCAAAGCCATTCTAG |
| UP_R_TCS13_LOX66 | CCTTATGGGATTTATCTTCCTTACATGTCTTAGTCCTCCAATG |
| UP_R_TCS14_LOX66 | CCTTATGGGATTTATCTTCCTTATAGAAAGCATCTTATTTCCC |
| UP_R_VICK_LOX66 | CCTTATGGGATTTATCTTCCTTAGCCAATACTAGTCATAATTC |
| UP_R_VNCRS_LOX66 | CCTTATGGGATTTATCTTCCTTAAGTATCTTCATACATTATCC |
| UPDELSTERCOVRS-A | TTGGAAATCCAGCTCCAGCACCGATATCC |
| UR_COMS | CCTTATGGGATTTATCTTCCTTATAAAACTCCTTTTAACTGTTAG |
| UR_SER | AGTAATTAAAAAGAAGATGG |
| UR_THR | TTGATTTATACCTCTCAATTT |

## REFERENCES


1. Haustenne L, Bastin G, Hols P, Fontaine L. 2015. Modeling of the ComRS signaling pathway reveals the limiting factors controlling competence in *Streptococcus thermophilus*. *Front Microbiol* 6:1413.

2. Fontaine L, Boutry C, Henry De Frahan M, Delplace B, Fremaux C, Horvath P, Boyaval P, Hols P. 2010. A Novel Pheromone Quorum-Sensing System Controls the Development of Natural Competence in *Streptococcus thermophilus* and *Streptococcus salivarius*. *J Bacteriol* 192:1444–1454.

3. Fontaine L, Goffin P, Dubout H, Delplace B, Baulard A, Lecat-Guillet N, Chambellon E, Gardan R, Hols P. 2013. Mechanism of competence activation by the ComRS signalling system in streptococci. *Mol Microbiol* 87:1113–1132.

4. Van Den Bogert B, Boekhorst J, Herrmann R, Smid EJ, Zoetendal EG, Kleerebezem M. 2013. Comparative genomics analysis of *Streptococcus* isolates from the human small intestine reveals their adaptation to a highly dynamic ecosystem. *PLoS One* 8: e83418.

5. Mignolet J, Fontaine L, Sass A, Nannan C, Mahillon J, Coenye T, Hols P. 2018. Circuitry Rewiring Directly Couples Competence to Predation in the Gut Dweller *Streptococcus salivarius*. *Cell Rep* 22:1627–1638.

6. Mignolet J, Cerckel G, Damoczi J, Ledesma-Garcia L, Sass A, Coenye T, Nessler S, Hols P. 2019. Subtle selectivity in a pheromone sensor triumvirate desynchronizes competence and predation in a human gut commensal. *Elife* 8:e47139.

7. Fontaine L, Dandoy D, Boutry C, Delplace B, de Frahan MH, Fremaux C, Horvath P, Boyaval P, Hols P. 2010. Development of a versatile procedure based on natural transformation for marker-free targeted genetic modification in *Streptococcus thermophilus*. *Appl Environ Microbiol* 76:7870–7787.

8. Fontaine L, Hols P. 2008. The inhibitory spectrum of thermophilin 9 from *Streptococcus thermophilus* LMD-9 depends on the production of multiple peptides and the activity of BlpGSt, a thiol-disulfide oxidase. *Appl Environ Microbiol* 74:1102–1110.

9. Liu X, Gallay C, Kjos M, Domenech A, Slager J, van Kessel SP, Knoops K, Sorg RA, Zhang J-R, Veening J-W. 2017. High-throughput CRISPRi phenotyping identifies new essential genes in *Streptococcus pneumoniae*. *Mol Syst Biol* 13:931.
