# Supplementary material for: The CovRS Environmental Sensor Directly Controls the ComRS Signaling System To Orchestrate Competence Bimodality in Salivarius Streptococci
Source: mBio. 2022 Jan 4;13(1):e03125-21. doi: 10.1128/mbio.03125-21 (PMC8725580; doi:10.1128/mbio.03125-21)
Supplement: TABLE S4 [file mbio.03125-21-st004.pdf]

Table S4. List of (overlapping) PCR fragments, synthetic DNA constructs, and EMSA probes

Strain/ Plasmid	PCR fragment	Template DNA	Primer 1	Primer 2
AK0001	Upstream homologous region (Up HR) of *tRNA*$_{thr}$ locus fused to P$_{comX}$-*luxAB*	JM1020	UF_THR	AK91
	spc, spectinomycin resistance cassette	pJUDspecmut1-gfp+ter	Up.fw.lox66	R_spec
	Downstream homologous region (Dw HR) of *tRNA*$_{thr}$ locus	HSISS4	LL61	UR_THR
AK0002 AK0003 AK0004 AK0005	Up HR of *tRNA*$_{ser}$ fused to *xylR*-P$_{xyl2}$	JM1125	UF_SER	RpxZ9_ATG
	cat, chloramphenicol resistance cassette	pJIMcat	Up.fw.lox66	Dn.rv.lox71
	Dw HR of *tRNA*$_{ser}$	HSISS4	LL150	DR_SER
AK0002	*comS* (HSISS4_10217)	HSISS4	MV23	MV25
AK0003	*xip*, mature C-terminal form of ComS (11aa)	HSISS4	MV24	MV25
AK0004	*comR* (HSISS4_00217)	HSISS4	MV29	MV30
AK0005	*comR*-P$_{comS}$-*comS* (HSISS4_00217-	HSISS4	MV29	MV25
AK0006	Up HR of *tRNA*$_{ser}$ fused to *xylR*-P$_{xyl2}$	AK0005	UF_SER	MV13
	xip-cat fused to Dw HR of *tRNA*$_{ser}$	AK0003	AK52	DR_SER
AK0007	Up HR of *tRNA*$_{ser}$	HSISS4	UF_SER	UR_SER
	cat, chloramphenicol resistance cassette	pJIMcat	AK69	AK70
	amiA3 (HSISS4_01366), Ami/Opp	HSISS4	AK44	AK71
	Dw HR of *tRNA*$_{ser}$	HSISS4	DF_SER	DR_SER
AK0008	Up HR of *amiACDEF* (HSISS4_01361-01365)	HSISS4	AK57	AK58
	xylR-P$_{xyl2}$	pZX10	AK59	AK50
	cat, chloramphenicol resistance cassette	pJIMcat	Up.fw.lox66	AK60
	Dw HR of *amiACDEF* (HSISS4_01361-01365)	HSISS4	AK61	AK62
AK0009	Up HR of *pptAB* (HSISS4_01518-01519)	HSISS4	AK133	AK509
	S. pneumoniae optimized P$_{lac}$ promoter	pJWV102-PL-dcas9	AK510	AK511
	S. pneumoniae optimized P$_{F6}$ promoter fused to the *lacI* repressor gene	pPEPY-PF6-lacI	AK512	AK337
	cat, chloramphenicol resistance cassette	pJIMcat	Up.fw.lox66	Dn.rv.lox71
	Dw HR of *pptAB* (HSISS4_01518-01519)	HSISS4	AK134	AK124
AK0010	Up HR of *tRNA*$_{thr}$	HSISS4	UF_THR	UR_THR
	Promoter of *comR* (P$_{comR}$)	HSISS4	AK55	AK56
	luxAB genes fused to *spc*-Dw HR *tRNA*$_{thr}$	AK0001	F_lux_ATG	DR_THR

AK0011<br>AK0012<br>AK0013	*cat*, chloramphenicol resistance cassette	pJIMcat	Up.fw.lox6<br>6	Dn.rv.lox7<br>1
	Dw HR of *covRS* (HSISS4_00246-00247)	HSISS4	AK188	AK189
AK0011	Up HR of *covR* and $covR_{D53A}$	HSISS4	AK184	AK246
	Dw HR of $covR_{D53A}$ and *covS* (HSISS4_00246-00247)	HSISS4	AK245	AK187
AK0012	Up HR of *covR* and $covR_{D53E}$	HSISS4	AK184	AK195
	Dw HR of $covR_{D53E}$ and *covS* (HSISS4_00246-00247)	HSISS4	AK194	AK187
AK0013	*covR* and Up HR of $covS_{T287A}$	HSISS4	AK184	AK252
	Dw HR of $covS_{T287A}$ (HSISS4_00247)	HSISS4	AK251	AK187
AK0014	Up HR of $tRNA_{thr}$	HSISS4	UF_THR	UR_THR
	P_{comX}-*opt-gfp*$^{+}$, *comX* promoter with 5'UTR optimized sequence fused to codon-optimized *gfp*$^{+}$	P_{comX}-*gfp*$^{+}$ (gBlocks DNA[a])	AK87	AK88
	spc fused to Dw HR of $tRNA_{thr}$	AK0001	Up.fw.lox6	DR_THR
AK0015	P_{xyl2}-*comR-cat* at $tRNA_{ser}$	AK0004	UF_SER	DR_SER
AK0016	Up HR of $tRNA_{ser}$ with upstream region of *xylR*	AK0004	UF_SER	AK107
	P_{xyl2}-*opt-gfp*$^{+}$, P_{xyl2} promoter with 5'UTR optimized sequence fused to codon-optimized *gfp*$^{+}$	P_{xyl2}-*gfp*$^{+}$ (gBlocks DNA[a])	AK108	AK88
	spc fused to Dw HR of $tRNA_{ser}$	JM1016	Up.fw.lox6	DR_SER
AK0017	P_{xyl2}-*comR-cat* at $tRNA_{thr}$	AK0004	UF_SER	DR_SER
AK0018	Up HR of *comS* (HSISS4_00217)	HSISS4	UF_R-lux	UR_comS
	Dw HR of *comS* (HSISS4_00217)	HSISS4	DF_comS	Dn_R<br>SS1-4
	cat, chloramphenicol resistance cassette	pJIMcat	Up.fw.lox6	Dn.rv.lox7
AK0019	Up HR of *amiACDEF* (HSISS4_01361-01365)	HSISS4	Opp_S4_6<br>B	Opp_S4_3<br>cat
	Dw HR of *amiACDEF* (HSISS4_01361-01365)	HSISS4	Opp_S4_2<br>cat	Opp_S4_1
	cat, chloramphenicol resistance cassette	pJIMcat	Up.fw.lox6	Dn.rv.lox7
AK0020	Up HR of *pptAB* (HSISS4_01518-01519)	HSISS4	AK121	AK122
	Dw HR of *pptAB* (HSISS4_01518-01519)	HSISS4	AK123	AK124
	erm, erythromycin resistance cassette	pGIUD0855er	AK169	AK170
AK0021	IPTG-inducible system of *pptAB* expression (P_{F6}-*lacI* P_{lac}-*pptAB*)	AK0009	AK133	AK124
AK0022	Up HR of $tRNA_{thr}$	HSISS4	UF_THR	UR_THR
	P_{comR}-*opt-gfp*$^{+}$, *comR* promoter with 5'UTR optimized sequence fused to codon-optimized *gfp*$^{+}$	P_{comR}-*gfp*$^{+}$ (gBlocks DNA[a])	AK112	AK88

	spc fused Dw HR of *tRNA*$_{thr}$	AK0001	Up.fw.lox66	DR_THR
AK0023	Up HR of *tRNA*$_{thr}$	HSISS4	UF_THR	UR_THR
	P$_{comS}$-*opt-gfp*$^{+}$, *comS* promoter with 5’UTR optimized sequence fused to codon-optimized *gfp*$^{+}$	P$_{comX}$-*gfp*$^{+}$ (gBlocks DNA[a])	AK106	AK88
	Spc fused-Dw HR of *tRNA*$_{thr}$	AK0001	Up.fw.lox66	DR_THR
AK0024	Up HR of *tRNA*$_{thr}$ locus fused to P$_{comS}$-*luxAB*	JM1019	UF_THR	AK91
	spc, spectinomycin resistance cassette	pJUDspecmut1-gfp+ter	Up.fw.lox66	R_spec
	Dw HR of *tRNA*$_{thr}$ locus	HSISS4	LL61	DR_THR
AK0025	P$_{xyl2}$-*comR* overexpression system fused to a chloramphenicol resistance cassette at the *tRNA*$_{ser}$ locus	AK0004	UF_SER	DR_SER
AK0023, AK0024	Up HR of *tnpII* locus	HSISS4	AK262	AK263
	spc, spectinomycin resistance cassette	pJUDspecmut1-gfp+ter	AK266	AK267
	Dw HR of *tnpII* locus	HSISS4	AK264	AK265
AK0023	*comS* under the xylose-inducible promoter P$_{xyl2}$	AK0002	AK88	AK448
AK0024	*xip* under the xylose-inducible promoter P$_{xyl2}$	AK0003	AK88	AK448
AK0025	Up HR of *tnpII* locus fused to a spectinomycin resistance cassette, *spc*	AK0023	AK262	AK153
	comS	HSISS4	AK151	AK152
	The constitutive promoter P$_{32}$	pJIMcat	AK362	AK222
	Dw HR of *tnpII* locus	HSISS4	AK264	AK265
AK0026	Up HR of *comR* together P$_{comR}$ and *comR* without stop codon	HSISS4	ML38	AK347
	gfp^{+}codon-optimzed gene followed by the *spc* resistance	AK0014	AK348	Dn.rv.lox71
	Dw HR of *comR* locus	HSISS4	ML40	ML41
AK0027	Up HR of *tRNA*$_{thr}$	HSISS4	UF_THR	UR_THR
	P$_{F6}$ synthetic constitutive promoter fused to the optimized *lacI* gene	pPEPY-PF6-lacI	AK336	AK337
	Dw HR of *tRNA*$_{thr}$ locus	HSISS4	Up.fw.lox66	DR_THR
AK0028	Up HR of *tRNA*$_{thr}$	HSISS4	UF_SER	UR_SER

	P_{lac} optimized promoter fused to a dead-cas9 (D10A H841A)	pJWV102-PL-dcas9	AK340	AK341
	Dw HR of *$tRNA_{thr}$* locus	HSISS4	Up.fw.lox66	DR_SER
AK0030	Up HR of *tnpII* locus	HSISS4	AK262	AK263
	P_{comX} promoter fused to the *luxAB* genes together with a spectinomycine resistance	AK0001	AK266	AK365
	Dw HR of *tnpII* locus	HSISS4	AK264	AK265
AK0031	Up HR of *tnpII* locus	HSISS4	AK262	AK263
	P_{comR} promoter fused to the *luxAB* genes together with a spectinomycine resistance	AK0010	AK266	AK505
	Dw HR of *tnpII* locus	HSISS4	AK264	AK265
AK0032	Up HR of *tnpII* locus	HSISS4	AK262	AK263
	P_{comX} promoter fused to the optimized *gfp^{+}* gene together with a spectinomycine resistance	AK0014	AK266	AK365
	Dw HR of *tnpII* locus	HSISS4	AK264	AK265
AK0036	*erm* resistance cassette fused to lox66 and lox71 sequences (*lox66-erm-lox71*)	pGIUD0855erm	ML45	ML46
	Up HR of *GOR* locus	HSISS4	AK458	AK452
	erm resistance cassette with lox66 and lox71 sequences with overlap flanking [illegible]	*lox66-erm-lox71*	AK455	AK462
	P_3 constitutive promoter fused to a guide targeting the *luc* gene together with a dcas9-handle and terminator	pPEPX-P3-sgRNAluc	AK463	AK464
	Dw HR of *GOR* locus	HSISS4	AK453	AK459
AK0037	Up HR of *tnpII* locus fused to a *spc* resistance cassette and the *luxAB* genes	AK0030	AK262	AK364
	P_{32} promoter	pJIMcat	AK361	AK362
	Dw HR of *tnpII* locus	HSISS4	AK264	AK265
AK0037	Up HR of *GOR* locus fused to the *erm* resistance, a terminator and the dcas9 handle	AK0036	AK458	AK472
	Dcas9 handle together with a guide targeting the P_{32} promoter	AK474	AK475	AK476
	P_3 promoter together with Dw HR of *GOR* locus	AK0036	AK473	AK459
AK0038, AK0039,	Up HR of *GOR* locus fused to the *erm* resistance, a terminator and the dcas9 handle	AK0036	AK458	AK472

AK0040	Dcas9 handle together with a guide targeting a modified version of the P_{covRS} promoter (P_{covRS*})	AK534	AK475	AK476
	P_3 promoter together with Dw HR of *GOR* locus	AK0036	AK473	AK459
AK0038, AK0039, AK0040	Up HR of *covRS* locus with 1 mutation in P_{covRS}	HSISS4	AK537	AK538
	covRS genes together with 1 mutation in P_{covRS}	HSISS4	AK539	AK187
	cat resistance cassette together with Dw HR of *covRS locus*	AK0011	Up.fw.lox66	AK544
AK0042	Up HR of LMD-9 *SUC* locus	LMD-9	AK565	AK566
	P_{comX}-*opt-gfp*$^{+}$, *comX* promoter with 5'UTR optimized sequence fused to codon-optimized *gfp*$^{+}$ followed by a *spc*	AK0014	AK569	AK570
	Dw HR of LMD-9 *SUC* locus	LMD-9	AK567	AK568
AK0042	Up HR of LMD-9 $tRNA_{ser}$ locus	LMD-9	AR901	PB11
	xylR-P_{xyl2} xylose inducible promoter	JM1125	AK147	AK576
	covR with D98 residue from LMG18311	LMG18311	AK575	AK612
	cat resistance cassette	pJIMcat	Up.fw.lox66	AK148
	Dw HR of LMD-9 $tRNA_{ser}$ locus	LMD-9	PB12	JB546
pBAD-covR-ST_{N-ter}	pBAD plasmid backbone	pBAD-comR-ST	AK287	AK291
	$covR_{WT}$	HSISS4	AK288	AK290
pBAD-$covR_{D53A}$-ST_{N-ter}	pBAD plasmid backbone	pBAD-comR-ST	AK287	AK291
	$covR_{D53A}$	AK0011	AK288	AK290
pBAD-$covR_{D53E}$-ST_{N-ter}	pBAD plasmid backbone	pBAD-comR-ST	AK287	AK291
	$covR_{D53E}$	AK0012	AK288	AK290
FV0002-FV0015	*cat*, chloramphenicol resistance cassette	JM1020	Up.fw.lox66	Dn.rv.lox71
FV0002	Up HR of *covS* (HSISS4_00247)	HSISS4	Up_F_covS	Up_R_covS_lox66
	Dw HR of *covS* (HSISS4_00247)	HSISS4	DN_F_covS_lox71	DN_R_covS
FV0003	Up HR of *ciaRH* (HSISS4_01096-01097)	HSISS4	Up_F_ciaRH	Up_R_ciaRH_lox66
	Dw HR of *ciaRH* (HSISS4_01096-01097)	HSISS4	DN_F_ciaRH_lox71	DN_R_ciaRH

FV0004	Up HR of *spaRK* (HSISS4_01036-01035)	HSISS4	Up_F_spaRK	Up_R_spaRK_lox66
	Dw HR of *spaRK* (HSISS4_01036-01035)	HSISS4	DN_F_spaRK_lox71	DN_R_spaRK
FV0005	Up HR of *vicK* (HSISS4_00837)	HSISS4	Up_F_vicK	Up_R_vicK_lox66
	Dw HR of v*icK* (HSISS4_00837)	HSISS4	DN_F_vicK_lox71	DN_R_vicK
FV0006	Up HR of *tcs06* (HSISS4_01178-01179)	HSISS4	Up_F_TCS06	Up_R_TCS06_lox66
	Dw HR of *tcs06* (HSISS4_01178-01179)	HSISS4	DN_F_TCS06_lox71	DN_R_TCS06
FV0007	Up HR of *bceRS* (HSISS4_01193-01192)	HSISS4	Up_F_bceRS	Up_R_bceRS_lox66
	Dw HR of *bceRS* (HSISS4_01193-01192)	HSISS4	DN_F_bceRS_lox71	DN_R_bceRS
FV0008	Up HR of *liaSR* (HSISS4_01345-01344)	HSISS4	Up_F_liaSR	Up_R_liaSR_lox66
	Dw HR of *liaSR* (HSISS4_01345-01344)	HSISS4	DN_F_liaSR_lox71	DN_R_liaSR
FV0009	Up HR of *tcs09* (HSISS4_00378-00379)	HSISS4	Up_F_TCS09	Up_R_TCS09_lox66
	Dw HR of *tcs09* (HSISS4_00378-00379)	HSISS4	DN_F_TCS09_lox71	DN_R_TCS09
FV0010	Up HR of *tcs10* (HSISS4_00352-00353)	HSISS4	Up_F_TCS10	Up_R_TCS10_lox66
	Dw HR of *tcs10* (HSISS4_00352-00353)	HSISS4	DN_F_TCS10_lox71	DN_F_TCS10_lox71
FV0011	Up HR of *vncRS* (HSISS4_01248-01249)	HSISS4	Up_F_vncRS	Up_R_vncRS_lox66
	Dw HR of *vncRS* (HSISS4_01248-01249)	HSISS4	DN_F_vncRS_lox71	DN_R_vncRS
FV0012	Up HR of *fasB* (HSISS4_01716)	HSISS4	Up_F_fasB	Up_R_fasB_lox66
	Dw HR of *fasB* (HSISS4_01716)	HSISS4	DN_F_fasB_lox71	DN_R_fasB
FV0013	Up HR of *tcs13* (HSISS4_01231-01230)	HSISS4	Up_F_TCS13	Up_R_TCS13_lox66
	Dw HR of *tcs13* (HSISS4_01231-01230)	HSISS4	DN_F_TCS13_lox71	DN_R_TCS13
FV0014	Up HR of *tcs14* (HSISS4_01447-1446)	HSISS4	Up_F_TCS14	Up_R_TCS14_lox66
	Dw HR of *tcs14* (HSISS4_01447-01446)	HSISS4	DN_F_TCS14_lox71	DN_R_TCS14
LF150<br>LF152<br>LF154	Up HR of LMG18311 *covRS* (stu0317-0318)	LMG18311	DD_UpDelcovRS-1	DD_UpDelcovRS-2
	cat, chloramphenicol cassette resistance	pJIMcat	Up.fw.lox66	Dn.rv.lox71
	Dw HR of LMG18311 *covRS* (stu0317-0318)	LMG18311	DD_DNDelcovRS-1	DD_DNDelcovRS-2

LF151	Up HR of LMG18311 *covRS* locus	LMG18311	SeqCovRS-F5	Up.insertCovRSkan-[illegible]
	kan resistance cassette	TOPO *kan*$^{R\ b}$	Kan OVL1	Kan OVL2
	Dw HR of LMG18311 *covRS* locus	LMG18311	Dn-insertcovRSKAN-F	DN-covRS-2
LF156	Up HR of LMD-9 *covRS* locus	LMD-9	SeqCovRS-F5	Up.insertCovRSkan-R
	kan resistance cassette	TOPO *kan*$^{R\ b}$	Kan OVL1	Kan OVL2
	Dw HR of LMD-9 *covRS* locus	LMD-9	Dn-insertcovRSKAN-F	DN-covRS-2
LF159	LMD-9 *covRS* locus	LMD-9	DD_UpDelcovRS-1	DD_DNDelcovRS-2
LF160	LMG18311 *covRS* locus	LMG18311	DD_UpDelcovRS-1	DD_DNDelcovRS-2
LF161	LMG18311 upstream *covRS* locus with D98E mutation introduction	LF151	UpdelstercovRS-A	LF-Up LMGCovR_D98E-
	LMG18311 upstream *covRS* locus with D98E mutation introduction and *kan* resistance cassette	LF151	LF-DN_LMGCovRD98E_3'A	DNcovRS-2
LF162	LMD-9 upstream *covRS* locus with E98D mutation introduction	LF156	UpdelstercovRS-A	LF-Up D9CovR_E98D-5'B
	LMD-9 upstream *covRS* locus with E98D mutation introduction and *kan* resistance cassette	LF156	LF-DN_D9CovR_E98D_[illegible]	DNcovRS-2
pMG36ET-covRS	*covRS* operon of LMG18311, to be restricted to perform ligation with restricted pMG36ET	LMG18311	LF-pMGcovRD9XbaI-F	LF-pMGcovSLMGPstI-R

**EMSA probes**	**Template**	**Primer 1**	**Primer 2**
P_{comR}-HSISS4 (*comR* promoter of HSISS4, non-fluorescent probe)	HSISS4	AK351	AK305
P_{comR}-HSISS4-Cy3 (*comR* promoter of HSISS4 with Cy3 dye at the 5' end)	HSISS4	AK304	AK305
P_{comR}-HSISS4-Cy3-Cy5 (*comR* promoter of HSISS4 with Cy3 and Cy5 dyes at the 5' and 3' end, respectively)	HSISS4	AK304	AK326

P*dnaE*-HSISS4 (*dnaE* promoter of HSISS4, non-fluorescent probe)	HSISS4	AK349	AK301
P*dnaE*-HSISS4-Cy3 (*comR* promoter of HSISS4, with Cy3 dye at the 5' end)	HSISS4	AK300	AK301
P*covR*-HSISS4-Cy3 (*covR* promoter of HSISS4, with Cy3 dye at the 5' end)	HSISS4	AK298	AK299
CDS*dnaE*-HSISS4 (150 pb fragment of the *dnaE* coding sequence of HSISS4, non-fluorescent probe)	HSISS4	AK350	AK303
CDS*dnaE*-HSISS4-Cy3 (150 pb fragment of the *dnaE* coding sequence of HSISS4, with Cy3 dye at the 5' end)	HSISS4	AK302	AK303
P*dnaE*-HSISS4$_{synth}$ (*dnaE* promoter of *S. salivarius*, with Cy3 dye at the 5'end)	P_{dnaE} (DNA_s)	AK354	AK355
P*comR*-HSISS4$_{synth}$ (*comR* promoter of *S. salivarius*, with Cy3 dye at the 5'end)	P_{comR} HSISS4 (DNA_s)	AK354	AK355
P*comR*-LMD-9$_{synth}$ (*comR* promoter of *S. thermophilus*, with Cy3 dye at the 5'end)	P_{comR} LMD-9 (DNA_s)	AK354	AK355
P_{comR}-NTC12167$_{synth}$ (*comR* promoter of *S. vestibularis*, with Cy3 dye at the 5'end)	P_{comR} NTC12167 (DNA_s)	AK354	AK355

[a]gBlocks™ DNA are synthetic dsDNA provided by Integrated DNA Technologies (IDT)
[b]TOPO© plasmid from Thermo-Fischer
